# Induction of RET Dependent and Independent Pro-Inflammatory Programs in Human Peripheral Blood Mononuclear Cells from Hirschsprung Patients

**DOI:** 10.1371/journal.pone.0059066

**Published:** 2013-03-18

**Authors:** Marta Rusmini, Paola Griseri, Francesca Lantieri, Ivana Matera, Kelly L. Hudspeth, Alessandra Roberto, Joanna Mikulak, Stefano Avanzini, Valentina Rossi, Girolamo Mattioli, Vincenzo Jasonni, Roberto Ravazzolo, William J. Pavan, Alessio Pini-Prato, Isabella Ceccherini, Domenico Mavilio

**Affiliations:** 1 Laboratory of Molecular Genetics, IRCCS, Giannina Gaslini Istitute, Genoa, Italy; 2 Department of Health Science, Biostatistics Unit, University of Genoa, Genoa, Italy; 3 Unit of Clinical and Experimental Immunology, Humanitas Clinical and Research Center, Rozzano, Milan, Italy; 4 Department of Medical Biotechnologies and Translational Medicine, University of Milan, Milan, Italy; 5 Department of Pediatric Surgery, IRCCS, Giannina Gaslini Institute, Genoa, Italy; 6 DINOGMI Department, University of Genova, Genova, Italy; 7 Mouse Embryology Section, Genetic Disease Research Branch, National Human Genome Research Institute, Bethesda, Maryland, United States of America; Charite Universitätsmedizin Berlin, Germany

## Abstract

Hirschsprung disease (HSCR) is a rare congenital anomaly characterized by the absence of enteric ganglia in the distal intestinal tract. While classified as a multigenic disorder, the altered function of the RET tyrosine kinase receptor is responsible for the majority of the pathogenesis of HSCR. Recent evidence demonstrate a strong association between RET and the homeostasis of immune system. Here, we utilize a unique cohort of fifty HSCR patients to fully characterize the expression of RET receptor on both innate (monocytes and Natural Killer lymphocytes) and adaptive (B and T lymphocytes) human peripheral blood mononuclear cells (PBMCs) and to explore the role of RET signaling in the immune system. We show that the increased expression of RET receptor on immune cell subsets from HSCR individuals correlates with the presence of loss-of-function RET mutations. Moreover, we demonstrate that the engagement of RET on PBMCs induces the modulation of several inflammatory genes. In particular, RET stimulation with glial-cell line derived neurotrophic factor family (GDNF) and glycosyl-phosphatidylinositol membrane anchored co-receptor α1 (GFRα1) trigger the up-modulation of genes encoding either for chemokines (CCL20, CCL2, CCL3, CCL4, CCL7, CXCL1) and cytokines (IL-1β, IL-6 and IL-8) and the down-regulation of chemokine/cytokine receptors (CCR2 and IL8-Rα). Although at different levels, the modulation of these “RET-dependent genes” occurs in both healthy donors and HSCR patients. We also describe another set of genes that, independently from RET stimulation, are differently regulated in healthy donors versus HSCR patients. Among these “RET-independent genes”, there are CSF-1R, IL1-R1, IL1-R2 and TGFβ-1, whose levels of transcripts were lower in HSCR patients compared to healthy donors, thus suggesting aberrancies of inflammatory responses at mucosal level. Overall our results demonstrate that immune system actively participates in the physiopathology of HSCR disease by modulating inflammatory programs that are either dependent or independent from RET signaling.

## Introduction

Hirschsprung disease (HSCR) is a rare and congenital anomaly of the enteric nervous system (ENS) that occurs with an average incidence of 1 into 5000 live births. It is characterized by the absence of enteric ganglia in variable lengths of distal intestinal tract, resulting from the premature arrest of the craniocaudal migration of vagal neural crest cells in the gut. HSCR has a complex genetic inheritance, characterized by low and sex-dependent penetrance with a strong male preponderance[1], [2].

The major gene involved in HSCR pathogenesis is *RET* (REarranged during Transfection), located on chromosome 10q11.2 and containing 21 exons, which codes for a tyrosine kinase receptor[3], [4]. Over one hundred RET mutations have been described throughout the gene, including large deletions, microdeletions, insertions, missense, nonsense and splicing mutations[5], [6], [7]. Overall, these genetic anomalies lead to loss of function of RET protein and/or to haploinsufficiency [8], [9], [10]. Although the majority of HSCR pedigrees show linkage with *RET*, the coding sequence mutations of the *RET* gene could be only identified in up to 50% of familial cases and in 7–20% of sporadic cases[11]. Therefore, non-coding mutations have been postulated to play a role in unexplained cases[12]. A haplotype, including two variants at −5 and −1 bp from the RET transcription starting site and a single nucleotide polymorphism (SNP) in exon 2 (c135G>A; A45A), was first identified as associated with the HSCR phenotype[13], [14], [15]. Furthermore, an intron 1 SNP (rs2435357 C>T) lying on the same haplotype was found to disrupt an enhancer site[12], [16] and to cause a reduced expression of the gene[17], [18]. Nevertheless, despite the fact that rs2435357 is considered as the predisposing HSCR mutation, additional functional data have shown a possible cooperative or synergistic role for other intronic variants and/or promoter polymorphisms[14], [19], [20].

RET protein is expressed in neural crest-derived cell lineages and is essential for their proliferation, migration and differentiation during embryonic development of kidneys, peripheral nervous system (sympathetic, parasympathetic and enteric) and for spermatogenesis[21], [22]. In this context, RET has been demonstrated to play a central role in several intracellular signaling pathways that regulate cellular survival, proliferation, differentiation, migration and chemotaxis[23]. The activation of the RET signaling cascade in physiological condition is secondary to the stimulation of the receptor through a multi-protein complex that involves both soluble ligands and cellular RET co-receptors. Indeed, this process first requires the specific binding of one of the four identified RET ligands [glial-cell line derived neurotrophic factor family (GDNF), neurturin, artemin, persephin] with one of the four glycosyl-phosphatidylinositol membrane anchored co-receptors (GFRα1-4)[24]. As a second step, this binary complex interacts with RET-receptor located in cell lipid rafts and the binding triggers a RET autophosphorilation of specific tyrosine residues[25]. Because of their pluripotent stem nature, neural crest cells expressing RET receptor migrate towards developing structures in the embryo to form tissues such as heart, bones and cartilage of the craniofacial compartment, peripheral and enteric neurons and glia and also skin's pigment cells and smooth muscle cells. Indeed, RET gene results expressed at variable levels in most of these tissues[26], [27], [28], [29].

*RET* transcripts have also been found in hematopoietic tissues such as fetal liver, thymus, spleen and lymph nodes, thus suggesting a role of *RET* in both the development and homeostasis of immune system[29], [30], [31], [32], [33], [34]. In this regard, it has been shown in a RET-deficient mouse model that this tyrosine kinase is a key regulator in the organogenesis of Peyer's Patches[35] and that RET receptor is also constitutively expressed on several human primary immune cells[31], [36]. Furthermore, RET/PTC1, a rearranged *RET* tyrosine kinase gene playing a causative role in the pathogenesis of papillary thyroid carcinoma, has been shown to induce a vast pro-inflammatory program, thus demonstrating that RET downstream signaling is directly associated with both inflammation and malignancies[37]. However, those studies examining the levels of RET receptor on human primary immune cells gave incomplete or controversial results without disclosing the definitive phenotypic distribution and levels of expression of this tyrosine kinase on adaptive and innate primary immune cells. Moreover, the functional correlates of RET receptor engagement on immune cells have not yet been disclosed.

The present study takes advantage of a large cohort of HSCR patients and control individuals to examine in detail the phenotypic distribution and the levels of RET receptor expression on primary immune cell subsets in correlation with RET genotypes and mutations. Moreover, to functionally dissect the physiology and physiopathology of this tyrosine kinase receptor in triggering immune responses, we analyzed the effects of RET engagement in modulating a large family of inflammatory/regulatory genes in the peripheral blood mononuclear cells (PBMCs) from both healthy donors and HSCR patients.

## Results

### Distribution and levels of expression of RET on immune cells

**Figure 1:**
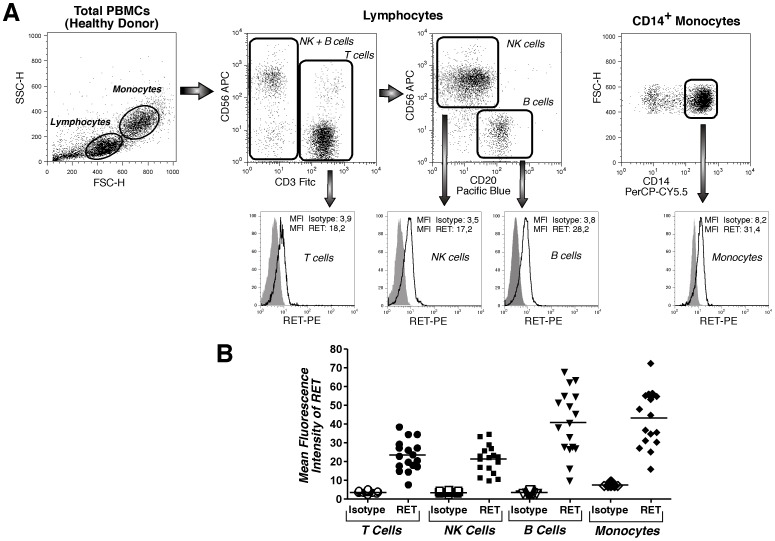
Phenotypic distribution and levels of expression of RET receptor on circulating immune cell subsets. (A) Flow cytometric dot plot (upper line) and histogram (lower line) graphs showing a representative example from an healthy donor of CD14^pos^ monocytes, CD56^pos^ (NK cells), CD3^pos^ (T cells) and CD20^pos^ (B cells) lymphocytes expressing RET receptor (black line) compared to isotype control (gray shaded histograms). (B) Summary graph of dot plots with medians (horizontal black bars) showing the mean fluorescence intensities (MFIs) of RET receptor on immune cells (black symbols) compared to that of isotype controls (open white symbols) from 17 healthy donors.

The first characterization of RET distribution within immune cell compartments performed in late 1990s did not detect any level of *RET* transcripts or RET protein in primary lymphocytes, differently from CD34^pos^ progenitor cells, macrophages and granulocytes that were found positive for RET expression[31]. This statement was implemented a few years later by another report that demonstrated the presence of several RET isoforms in monocytes, T (both CD4^pos^ and CD8^pos^ subsets) and in B cells[36]. In particular, this study detected for the first time the expression of RET receptor on the cell membrane of both lymphocytes and monocytes through an indirect and single-color flow cytometric approach. However, neither percentages of expression nor mean fluorescence intensities (MFIs) exactly quantified the levels of RET expressed on immune cell subsets. To complete the phenotypic distribution of RET receptors on cells from immune system, we extensively analyzed the expression of this tyrosine kinase on human PBMCs of healthy donors through a multicolor flow cytometric approach. Our results confirmed a clear expression of RET receptor on both lymphocytes and monocytes. Moreover, our data showed that, other than CD14^pos^ monocytes, CD3^pos^ T and CD20^pos^ B cells, also CD56^pos^ Natural Killer (NK) cells express remarkable levels of RET receptor (Figure 1A). These results were also confirmed by experiments of confocal microscopy (Figure S1). Indeed, the MFIs of RET on all cell subsets were significantly higher when compared with matched isotype controls (Figure 1B), and the overall expression of RET receptor appeared to be different and highly variable among the immune cell compartments analyzed.

**Figure 2:**
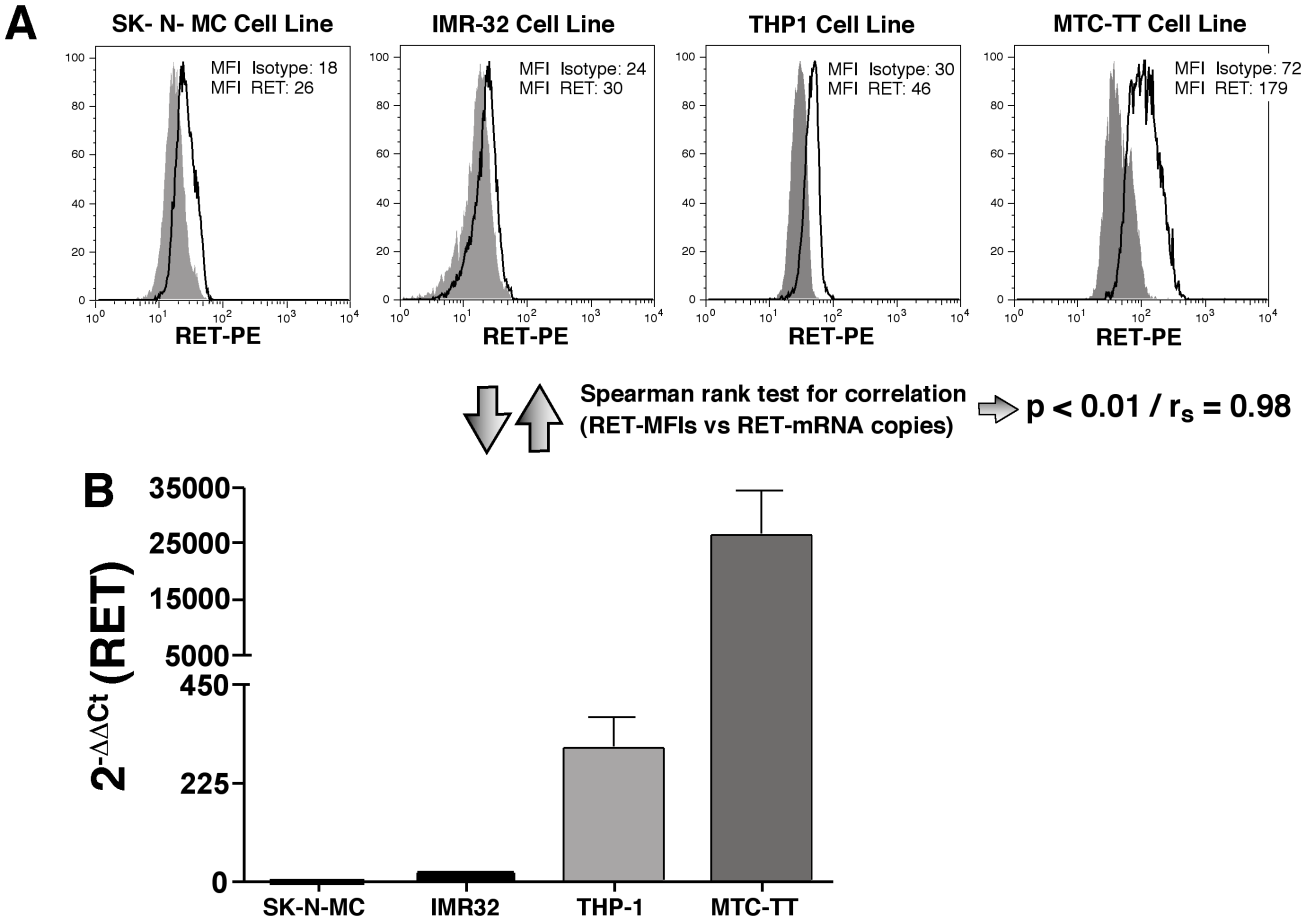
Correlation between the RET receptor expression and RET transcripts on four different cell lines. (A) Flow cytometric histogram graphs showing the MFI levels of expression of RET receptor (black line) of 4 different cell lines. Gray shaded histograms represent the isotype controls. (B) Histogram bar graph showing the number of RET mRNA copies produced by the same cell lines displayed in panel A and analyzed within the same time-frame in culture. Values are normalized on SK-N-MC cell line of one experiment and are reported as fold increased in expression (2^−ΔCt^) as mean of three independent experiments. Of note, the level of RET receptor expressed on cell membrane significantly correlated with the amount of RET transcripts, as assessed by the Spearman rank test for correlation.

To assess whether the levels of expression of RET on PBMCs detected by the above-mentioned flow-cytometric approach were reliable and could be used to exactly quantify the amount of receptor presents on cells, we then proceeded to verify the specificity and sensitivity of the anti-RET monoclonal antibody (mAb) by analyzing the expression of RET receptor on several cells lines known to express either high or low levels of this gene (Figure 2A). As expected, we could detect very low levels of RET receptor on IMR-32 and SK-N-MC cells, two neuroblastoma cell lines shown to have small amount of RET transcripts[17], [38]. In contrast, we found that RET receptor was expressed at very high levels on the membrane of MTC-TT cell line, which derives from a medullary thyroid carcinoma (MTC) associated with multiple endocrine neoplasia type 2A (MEN2A) and that produces high levels of RET mRNA copies[39]. Finally, we detected substantial levels of RET receptor on THP1 cell, a monocytic leukemia cell line also known to produce remarkable amount of RET transcript[31], although at intermediate level if compared with our negative (IMR-32 and SK-N-MC cell lines) and positive (MTC-TT cell line) controls. The different levels of RET receptor expressed on the membrane of these cell lines showed a statistical significant correlation with their relative copies of RET mRNA transcript (Figure 2B), thus demonstrating that the levels of expression of RET receptor exactly matched with the amount of RET mRNA transcribed within the same cell lines. All together, these results confirm that our flow cytometric approach is a reliable methodology to detect the presence and levels of expression of RET receptor on cell membrane.

### Expression of RET on immune cells from HSCR patients and associations with RET genotypes

**Figure 3:**
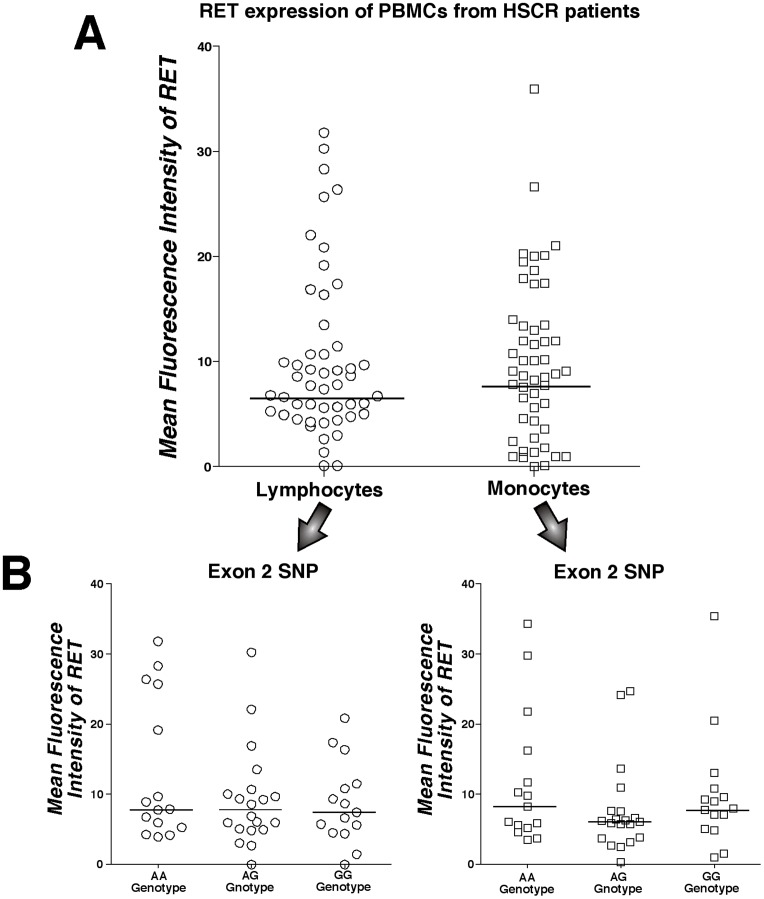
Expression of RET receptor on immune cells from HSCR patients associated with a single nucleotide polymorphism at exon 2. Summary graph of statistical dot plots showing MFI values of RET receptor expressed on lymphocytes and monocytes from 50 HSCR patients with medians (horizontal black bars) either in the absence (A) or in the presence (B) of an association analysis stratification based on the genotype at the exon 2 of RET gene (SNP rs1800858). We did not detect any statistically significant association between the phenotypic distribution of RET receptor on immune cells with RET genotype at exon 2 SNP.

Since loss-of-function RET mutations are pathogenic allele variants leading to the onset of HSCR, we analyzed the expression of RET receptor on immune cell subsets from a cohort of 50 HSCR patients. As observed for healthy donors, we found that the overall amounts of RET receptor on lymphocytes and monocytes from HSCR patients were comprised in a large range of expression, thus demonstrating a large degree of inter-individual variations (Figure 3A). Moreover, we did not detect any specific phenotypic distribution of RET receptor (neither increased nor decreased expression) among the several subsets of primary immune cells analyzed (data not shown).

We next determined if the expression of RET receptor on immune cells from HSCR donors correlates with specific genotypes at the RET locus. In particular, we focused on four single nucleotide polymorphisms (SNPs) of the coding region located at exons 2 (rs1800858), 11 (rs1799939), 13 (rs1800861) and 14 (rs1800862), that are known to have different frequencies in HSCR patients versus normal controls[40]. Exon 2 SNP represents a tag marker for the HSCR predisposing haplotype[16] and it has been shown to be associated with a reduced gene expression[17] **.** Therefore, a distorted distribution of the two alleles of the exon 2 SNP would theoretically explain the variability of RET receptor expression on PBMCs from HSCR patients. To test this hypothesis, the cohort of HSCR subjects was subdivided into the three subgroups having different genotypes at RET exon 2 SNP locus, namely GG (present on 15 HSCR patients), GA (present on 20 HSCR patients) and AA (present on 15 HSCR patients), with GG and AA being the wild type and homozygote variants, respectively. Our analyses did not reveal any significant difference of RET receptor expression between the three subgroups of lymphocytes and monocytes from HSCR patients showing different RET genotypes at this locus (Figure 3B). Even when lymphocyte subsets (B, T and NK cells) were separately analyzed, we did not observe any differential effects of the RET genotype at the exon 2 SNP locus on the expression of RET receptor (Figure S2). These data demonstrate that neither the presence nor the absence of the HSCR predisposing haplotype affects the phenotypic distribution of RET on circulating immune cells. Finally, we also evaluated the presence of a possible association between the levels of the RET receptor and the genotypes at the three other SNPs analyzed, namely those of exons 11, 13 and 14. Similar to the results obtained for exon 2 SNP locus of RET gene, no significant associations were observed between these latest genotypes and the expression of RET on immune cells from HSCR patients (Figure S3).

**Figure 4:**
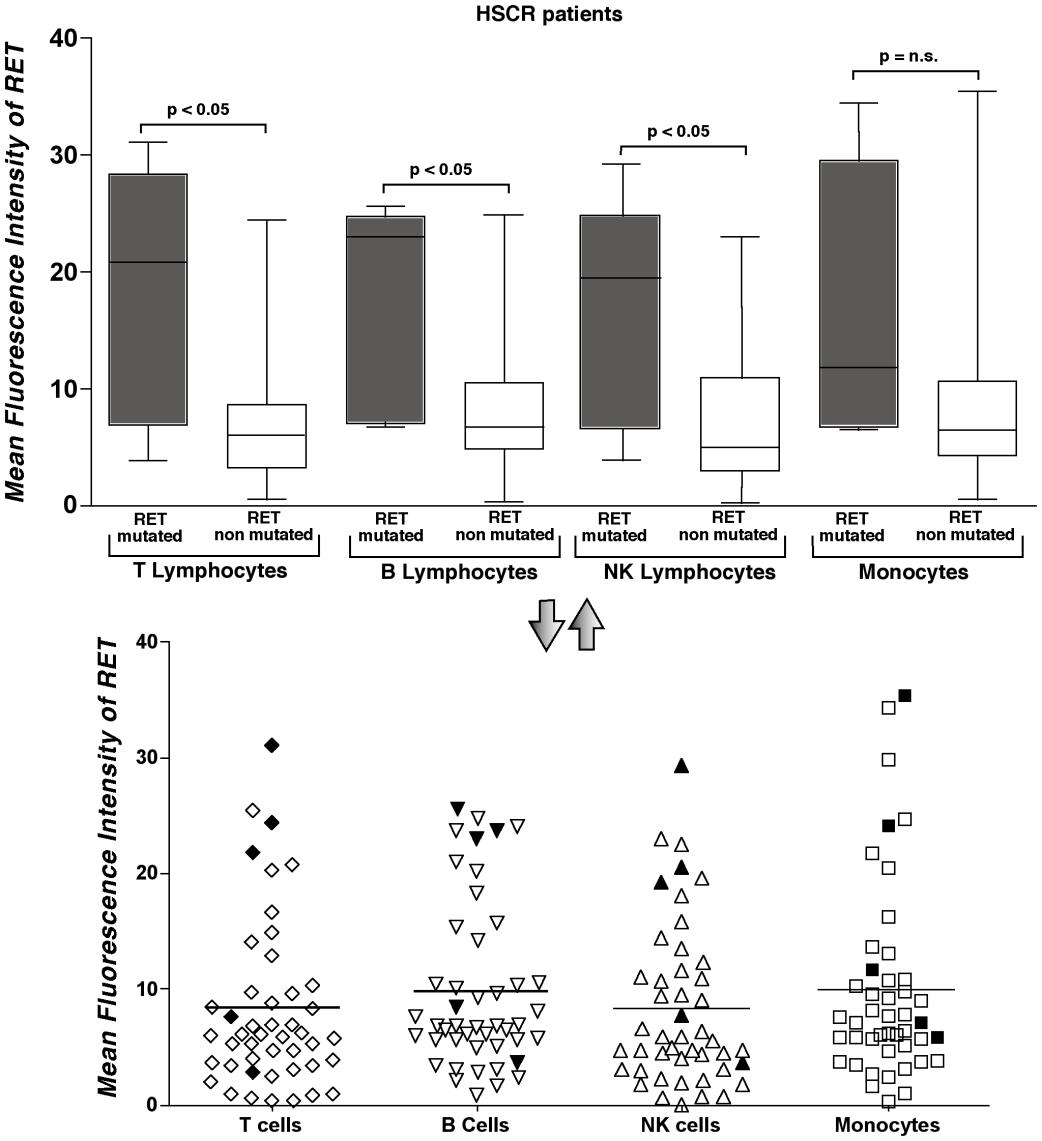
Expression of RET receptor on immune cells from HSCR patients associated with pathogenic mutations of RET gene. Summary graph of statistical histogram bars (upper panel) and dot plots (lower panel) showing MFI values of RET expressed on monocytes, T, B and NK lymphocytes from 46 HSCR patients stratified on the basis of individuals either carrying (gray histogram bars in upper panel and black symbols in the lower panel) or not carrying (empty histogram bars in upper panel and empty symbols in the lower panel) pathogenic RET mutation. 4 HSCR patients were excluded from the analysis because they were carrying mutations of the RET gene with uncertain effects.

After having excluded any possible role of RET genotypes in modulating the expression of RET receptor on immune cells, we evaluated whether RET gene mutations in cells from HSCR patients affected the expression of RET receptor. Mutational analysis of *RET* gene within the cohort of 50 HSCR donors identified 9 distinct RET sequence variations. We then proceeded to distinguish between potentially causative mutations and neutral variants of *RET*. To this end, the impact of these variations on protein structure and function for non-synonymous changes and on splicing for intronic or synonymous variants was evaluated using Polyphen, SIFT and Panther softwares (Table S1)[41], [42], [43], [44], [45], [46]. Data already published on functional assays of RET mutations were also used to recognize putative severe nucleotide changes[47]. Five of the nine variants demonstrated a significant “pathogenic” status, while the impact of other 4 gene variants on RET function was uncertain. Among these latter four mutations, there are the intronic variants c.1063+9G>A and c.1880-16C>T, the sequence c.2944C>T (p.R982C) whose role in determining HSCR had already been excluded by functional tests[8] and the c.2556C>G (p.I852M) variation which has been considered as a tolerable mutation by all the algorithms. These four non-pathogenic mutations were no longer considered for our analyses. We then assessed whether the amounts of RET receptor on immune cells was affected by the presence of the 5 pathogenic mutations. Our data show that the levels of RET receptor expression were significantly higher on all lymphocyte subsets (B, T and NK cells) of HSCR patients carrying a putative pathogenic variant of RET gene compared to those of HSCR patients not carrying any mutation in RET gene. Although not statistic significant, we observed a similar trend also within the monocyte compartment (Figure 4).

Similar to what we found by comparing immune cells from HSCR individuals with and without mutations of RET gene, we detected a statistically significant higher expression of RET receptors in PBMCs from HSCR patients with loss-of-function RET pathogenic variant compared to that of HSCR individuals carrying a non-pathogenic mutation of the same gene. Indeed, the levels of RET receptor on PBMCs from this latter cohort of patients were similar to that of HSCR patients without any known RET gene mutation (Figure S4). This finding implies that only the putative pathogenic RET gene variants are associated with the increased expression of RET receptor on immune cells.

### RET dependent and independent pro-inflammatory programs in immune cells from HSCR patients and healthy donors

Although it has been demonstrated that the activation of RET signaling pathway induces the expression of a large set of pro-inflammatory genes in primary thyrocytes[37] and that RET is actively involved in immune system homeostasis[35], very little is known on the physiologic role of RET receptor expressed on circulating immune cells. Our hypothesis is that the engagement of RET receptor expressed on primary immune cells is somewhat involved in the homeostasis of immune responses and that the stimulation of this tyrosine kinase on PBMCs from HSCR patients might be possibly associated with some severe and inflammatory clinical outcomes, such as Hirschsprung's associated enterocolitis (HAEC). To test this idea, we customized a TLDA “inflammatory” card containing the primers and probes of 96 genes that included all main cytokines, chemokines, receptors and immune mediators normally involved during acute and chronic phases of inflammation (Table S2).

In order to trigger the RET pathway on PBMCs, we incubated RET^pos^ PBMCs with GDNF ligand (100 ng/ml) and the GFRα1 co-receptor (1 µg/ml**)** for 5 hours on the basis of both our experience and previous published reports[36], [48]. Through this experimental setting we were able to examine the effects of RET engagement in modulating the transcript levels of all 96 customized genes comprised within “inflammatory” TLDA card, which also included 5 reference genes (GAPDH, actin, 18s ribosomal RNA, β2-microglobulin and HPRT1) (Table S2). The evaluation of the transcript levels in PBMCs from healthy donors and HSCR patients, either in the presence or in the absence of treatment with GDNF and GFRα1, revealed that 78 out of 96 genes were expressed (Table S3). We next proceeded to analyze the statistics of those 69 genes for which there were no more than two data missing in one of the four groups (treated and untreated PBMCs, healthy donors and HSCR patients) or one data missing in more than one group.

**Figure 5:**
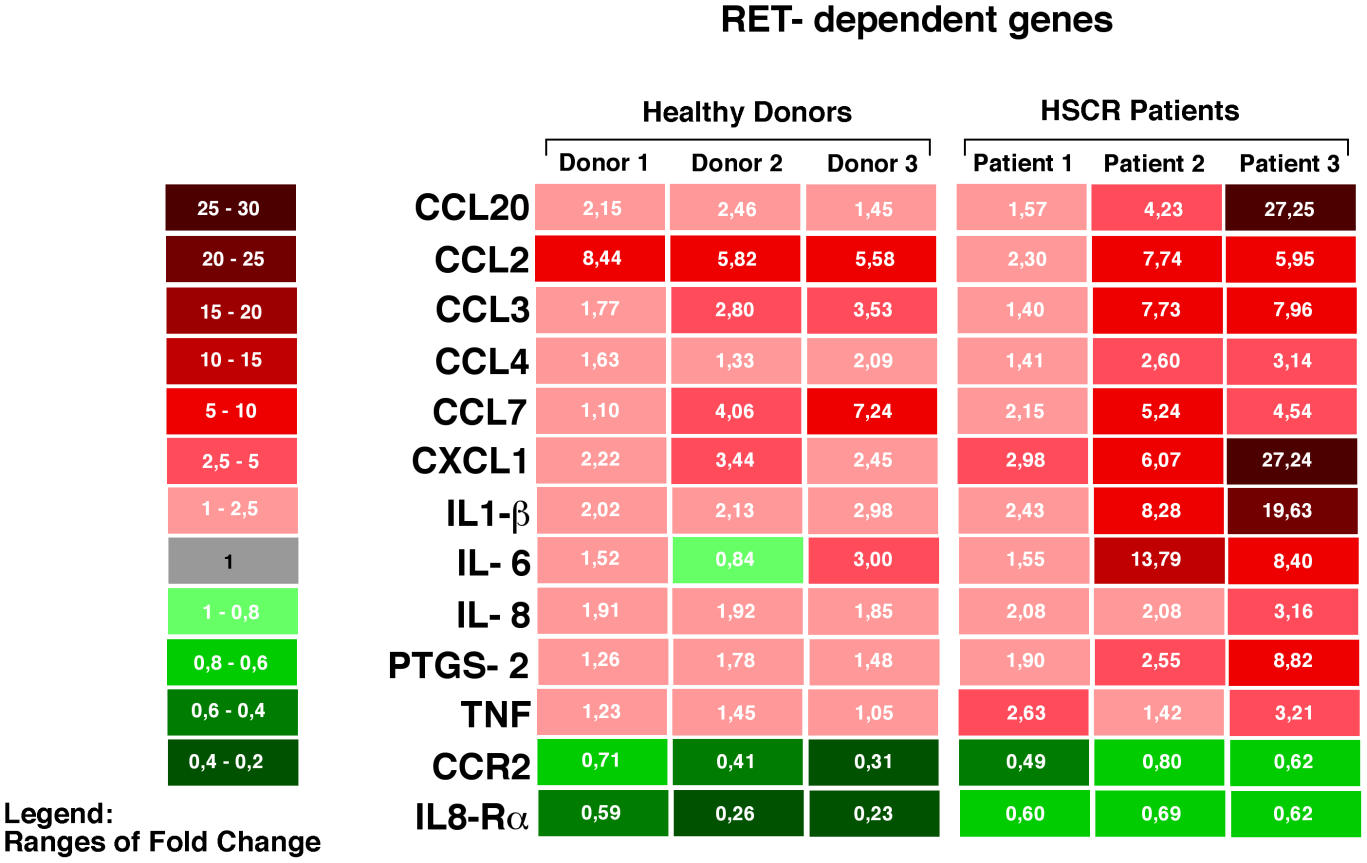
Modulation of RET-dependent genes. Colorimetric scale graph showing the fold increase (red) and decrease (green) of mRNA transcripts for those genes modulated in both PBMCs from healthy donors and HSCR patients following treatment with GDNF and GFRα1. The differences in fold changes of RET-dependent genes between the PBMCs of three healthy donors and three HSCR patients analyzed are indicated by means of 2^−ΔΔCt^ values included in each square and by the ranges of color tonality of the same square.

Our analyses identified two main clusters of genes exhibiting different modulatory patterns (Table S3). The first group includes 13 genes that displayed differences in their transcript levels following RET stimulation. The altered expression of genes in this group appear to be “RET-dependent”, as the engagement of RET with GDNF and GFRα1 induced a significant modulation of these genes in both healthy donors and HSCR patients. Among this group 1, there are different chemokines (CCL2, CCL3, CCL4, CCL7, CCL20, CXCL1), cytokines (IL-1β, IL-6 and IL-8), one chemokine receptor (CCR2), one cytokine receptor (IL8-Rα), the tumor necrosis factor (TNF) and the prostaglandin-endoperoxide synthase 2 (PTGS2). Among all these 13 RET-dependent genes, CCR2 and IL8-Rα resulted to be down-modulated in healthy donors and HSCR patients, while all other genes were found to be up-regulated in both cohorts. We then proceeded to analyze whether the activation of RET signaling pathway with its ligand and co-receptor induced a different regulation of these genes in healthy donors compared to HSCR patients. Although the differences did not reach a statistical significance, the transcripts of the majority of the13 RET-dependent genes were expressed at higher levels in PBMCs from HSCR patients compared to healthy donors, with the only exception of CCL2 and CCL7. Even the fold decrease of CCR2 and IL8-Rα was lower in PBMCs from HSCR patients compared to that of healthy donors (Figure 5).

In order to confirm the results obtained with the TLDA “inflammatory” customized card, we enlarged our cohorts by enrolling 3 additional healthy donors and 3 HSCR patients and we proceeded to validate the modulation of the 13 RET-dependent genes by incubating PBMCs with GDNF and GFRa1 co-receptor and by performing an alternative real time PCR-based experimental approach. The results showed a very similar pattern of regulation and a similar heterogeneity in both the 3 additional healthy donors and 3 HSCR patients. Indeed, our analysis confirmed that 12 out of the 13 RET-dependent genes responded to the treatment by changing their expression in the same direction indicated by the TLDA array, with the only exception of the TNF gene. In particular, 10 genes were up-regulated upon RET activation, while CCR2 and IL8 Rα were down-regulated (Table S4). Although only the CCL20 and PTGS2 genes showed a statistically different expression between treated and untreated cells, the general trend of the13 RET-dependent gene expression was maintained before and after the treatment, similar to what we observed with TLDA array (p = 0.0001 at the binomial test).

In order to further strengthen our results, we also tested whether the up-regulation of several genes following the engagement or RET was associated with an increased secretion in cell supernatants of the related soluble and inflammatory cytokines and chemokines. Regardless of the treatment of PBMCs with RET ligand and co-receptor, we could not detect any levels of IL-8, CCL4, TNF and CCL7, in line with the fact that their related genes were the ones showing the lowest fold increases among the13 RET-dependent genes. In contrast, we found that the engagement of RET receptor with its ligand and co-receptor resulted in an increased production of all other measured cytokines and chemokines. We also found that the heterogeneous up-modulations of the RET dependent genes was also mirrored by a similar heterogeneity in the levels of the relative cytokines and chemokines secreted in cell supernatants by PBMCs. Even the different degrees of positive regulation of CCL20, IL1-β, CCL2, CCL3, IL-6, CXCL1 between healthy donors and HSCR patients paralleled the amounts of related soluble molecules present in cell culture. Indeed, taking CCL3 and CXCL1 as representative examples, we could not detect any production of these two chemokines from PBMCs of healthy donors in line with low levels of up-modulation of their related genes in this cohort. On the other side, the engagement of RET induced a higher secretion of detectable amounts of CCL3 and CXCL1 in HSCR patients, in line with higher levels of positive regulation of their related genes (Table 1).

**Table 1:** Levels (pg/ml) and standard deviation (SD) of soluble inflammatory cytokines and chemokines measured in the supernatant of PBMCs either in the absence (italic) or in the presence (bold italic) o treatment with GDNF and GFRα1.

	CCL20	IL-1β	CCL2	CCL3	CCL7	IL-6	CXCL1	TNF	CCL4	IL-8
Healthy Donor 1/Untreated	Undetectable	Undetectable	Undetectable	Undetectable	Undetectable	Undetectable	Undetectable	Undetectable	Undetectable	Undetectable
Healthy Donor 1 Treated	49.5 (SD±0.7)	Undetectable	46.5 (SD±3.5)	Undetectable	Undetectable	Undetectable	Undetectable	Undetectable	Undetectable	Undetectable
Healthy Donor 2 Untreated	31.5(±0.70)	Undetectable	Undetectable	Undetectable	Undetectable	Undetectable	Undetectable	Undetectable	Undetectable	Undetectable
Healthy Donor 2 Treated	131.5 (SD±0.70)	16 (SD±0.2)	Undetectable	Undetectable	Undetectable	Undetectable	Undetectable	Undetectable	Undetectable	Undetectable
Healthy Donor 3 Untreated	Undetectable	Undetectable	25.37 (SD±1.8)	Undetectable	Undetectable	Undetectable	Undetectable	Undetectable	Undetectable	Undetectable
Healthy Donor 3 Treated	30(±0.0)	Undetectable	30.26 (SD±3.85)	Undetectable	Undetectable	31.26 (SD±2.12)	Undetectable	Undetectable	Undetectable	Undetectable
										
HSCR Patient 1 Untreated	Undetectable	Undetectable	Undetectable	28.55 (SD±6.15)	Undetectable	35.76 (SD±4.68)	38.93 (SD±6.68)	Undetectable	Undetectable	Undetectable
HSCR Patient 1 Treated	469 (SD±52.32)	226.5 (SD±2.12)	Undetectable	74.45 (SD±7.14)	Undetectable	89.03 (SD±10.41)	191.47 (SD±33.99)	Undetectable	Undetectable	Undetectable
HSCR Patient 2 Untreated	Undetectable	Undetectable	Undetectable	Undetectable	Undetectable	Undetectable	Undetectable	Undetectable	Undetectable	Undetectable
HSCR Patient 2 Treated	21 (SD±1.4)	Undetectable	Undetectable	Undetectable	Undetectable	Undetectable	Undetectable	Undetectable	Undetectable	Undetectable
HSCR Patient 3 Untreated	38.3 (SD±2.12)	9.5 (SD±2.12)	17.27 (SD±0.4)	Undetectable	Undetectable	39.74 (SD±10.77)	Undetectable	Undetectable	Undetectable	Undetectable
HSCR Patient 3 (Treated)	72.5 (SD±0.70)	422 (SD±35.4)	51.39 (SD±2.4)	32.91 (SD±9.3)	Undetectable	198. 89 (SD±19.02)	68.35 (SD±6.57)	Undetectable	Undetectable	Undetectable

**Figure 6:**
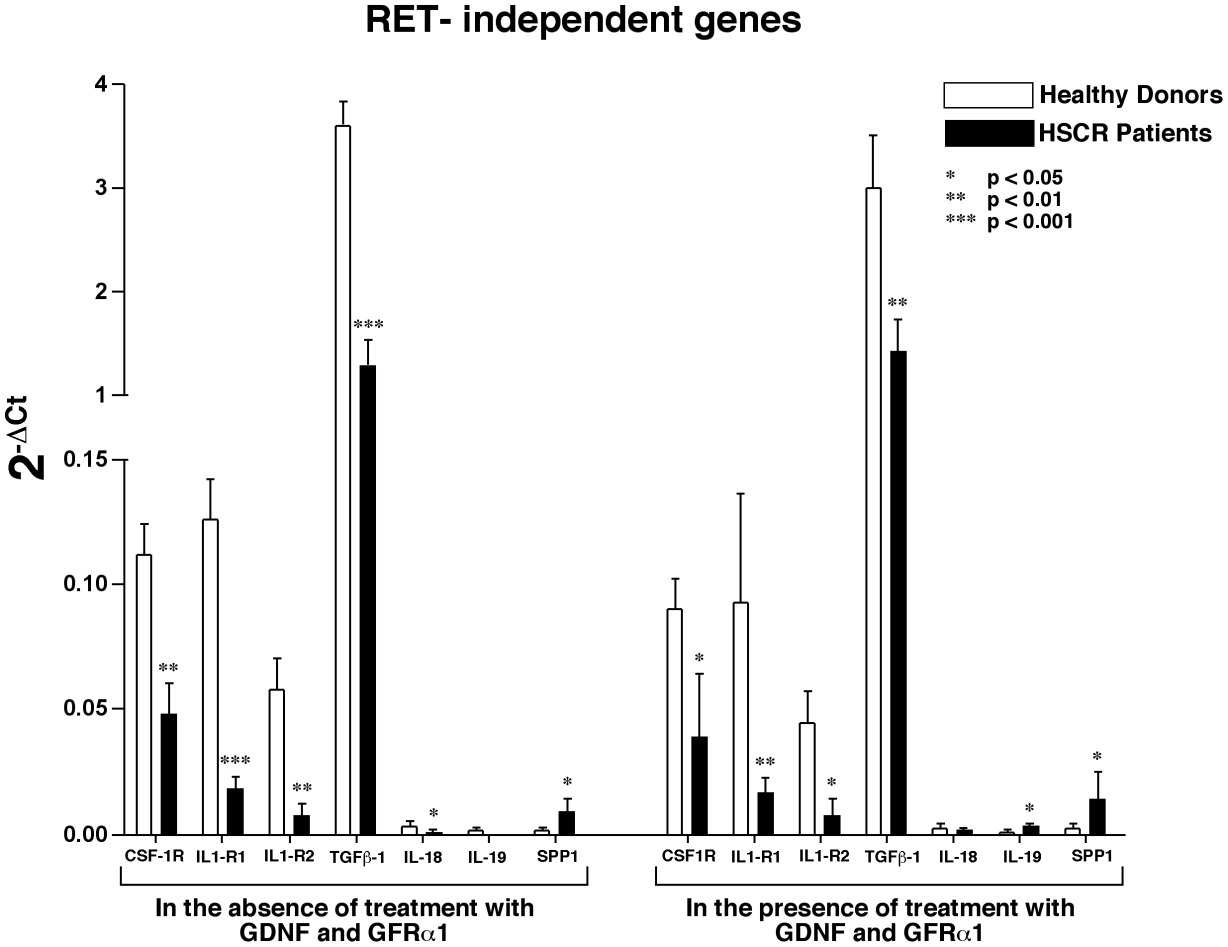
Modulation of RET-independent genes. Histogram bar graph showing the relative transcript levels of genes that, regardless of treatment with GDNF and GFRα1, are differently modulated in PBMCs from healthy donors and HSCR patients. The amounts of mRNA either in freshly purified (left part of the graph) or treated (right part of the graph) of PBMCs of healthy donors (white bars) and HSCR patients (black bars) were detected by Taqman Low Density Array (TLDA) card.

The second cluster is composed of genes that are differently expressed in healthy donors compared to HSCR patients and it can be classified as either “RET-independent” or “HSCR associated”, since the modulation of transcripts levels was not associated with RET engagement but seemed to be intrinsic to the HSCR itself. This second group includes genes encoding for the colony stimulating factor 1 receptor (CSF-1R), two members of the family of the interleukin 1 receptor (IL1-R1 and IL1-R2), transforming growth factor beta 1 (TGFβ-1), interleukin 18 and 19 (IL18 and IL19) and the secreted phosphoprotein 1 (SPP1). In particular, our analyses showed that modulation of CSF-1R, IL1-R1, IL1-R2, TGFβ-1 and IL-18 genes were significantly higher in both freshly purified and treated (with GDNF and GFRα1) PBMCs from healthy donors compared to that HSCR patients. In contrast, the mRNA transcripts of IL-19 and SPP1 resulted higher in HSCR patient compared to healthy donors (Figure 6).

We also identified a third set of genes (Group 3 in Table S3) whose levels of mRNA transcripts were differently regulated within each donor in both cohorts of healthy controls and HSCR patients regardless of treatment of PBMCs with GDNF and GFRα1. Due to the heterogeneous expression of genes included in this Group 3, it was not possible to perform statistic analyses and draw definite clear conclusions. Finally, a last set of genes whose changes are not statistically significant was also identified and no longer considered [labeled as not significant (n.s.) in Table S3].

## Discussion

The RET gene is an established key factor needed for the development of both the enteric nervous and the escretory system[49], [50] and its loss of function mutations leads to the onset of HSCR[5], [6], [7]. Recently, it has been demonstrated that RET is expressed on primary immune cells and also plays a major role in the regulation of both immune system homeostasis and inflammation[35], [36], [37]. In the present study, we took advantage of a unique cohort of HSCR and healthy donors to fully explore the cellular distribution and the levels of RET receptor on human PBMCs. In particular, we characterized and quantified the levels of expression of RET receptor on human monocytes, B, T, and NK cells. To this end, we first correlated the amounts of RET transcripts with the relative levels of RET protein expressed on several cell lines known to transcribe either high or low copies of RET mRNA (i.e. IMR-32, SK-N-MC, MTC-TT and THP1 cell lines) [17], [31], [38], [39]. Our results show a significant correlation between the two above-mentioned parameters, thus clearly demonstrating that our multicolor flow-cytometry experimental approach is a reliable methodology to exactly quantify the MFI of RET expression on cell membrane and can be also an useful tool for others in the field.

We then evaluated the levels of RET receptor on a large cohort of HSCR patients and we found an heterogeneous level of expression of RET protein within all four compartment of innate (monocytes and NK cells) and adaptive (T and B lymphocytes) immune cells analyzed. To better understand this phenomenon, we stratified the patients on the basis of both their RET genotypes and mutations, given the fact that specific RET haplotypes, such as the one containing the SNP2 variant allele, are associated with reduced RET expression[16]. Our results excluded any possible role of the exon 2 marker in determining the levels of RET receptor, thus not confirming that the reduced RET mRNA transcript are associated with the predisposing haplotype, as previously reported[17], [18]. This discrepancy might be explained by postulating differences in the tissue specific pattern of RET expression or between the mRNA and protein expression levels. As an example, these haplotypes may exert effects on the neural crest and kidney developmental defects but may not have effects on RET receptor levels and functions in the immune system components. We had similar negative data also for the other three additional RET coding SNPs in exons 11, 13 and 14.

Genomic analysis of the RET locus was also used to determine which HSCR patients had deleterious mutations that may influence the expression of RET receptor on immune cells. Nine out of fifty patients carried potentially pathogenic RET mutations and the effect of these variants was predicted by means of several different software (Table S1). Only 5 out of 9 mutations were pathogenic while the remaining 4 variants were not further considered due to the lack of clear evidence about their pathogenic effect. We then associated the five remaining pathogenic mutations, predicted to have severe effects on the functional outcome of RET protein, with the levels of RET receptor on primary immune cells. Surprisingly, we found that T, B and NK cells from HSCR patients carrying the RET loss-of-function mutations express significantly higher amounts of RET receptor compared to those of HSCR individuals not carrying pathogenic RET variants. Monocytes showed a similar trend, although it did not reach a statistical significance. In this regard, we have to take into account that only 20% or less of sporadic HSCR patients carry loss-of-function RET variants[12], [40], thus explaining why we could find only 5 of these patients in our large cohorts of 50 HSCR individuals. We are currently recruiting others HSCR subjects carrying pathogenic RET variants in order to increase the power of our statistics also within the monocyte compartment. This unexpected higher expression of RET receptors on lymphocytes from HSCR patients carrying severe RET mutations might be explained by two different cellular mechanisms: 1) a transcriptional feed-back assuring proper levels of the RET protein in the presence of low RET activity through a compensatory mechanism already described also for other mutant disease genes [51], [52] and 2) an intracellular accumulation of mutant RET receptor due to protein misfolding, as already reported for other defective cell membrane receptors leading to impairment of the cell response mechanisms [i.e. the ubiquitin- proteasome system (UPS) or autophagy] and inducing the onset of human disease[53], [54]. Further analysis will be needed to confirm such an observation, and eventually to test these two hypotheses.

Another aim of the present work was to assess whether the presence of RET receptors on primary immune cells is functionally relevant in activating inflammatory process, as demonstrated for primary thyrocytes[37]. To this end, we customized a 96 real-time PCR array, namely an Array Card with inventoried TaqMan® Gene Expression Assays, in which the inflammation pathways tested are represented by all main cytokines and chemokines together with their related receptors, and by other immune mediators normally involved in the inflammatory processes. Through this customized “inflammatory card”, we sought to investigate the expression patterns of specific target genes both in physiological and pathological conditions by assaying cells from controls and HSCR patients. In fact, before RNA extraction, cells were treated with the GDNF and GFRα1, whose expression in human PBMCs had previously been excluded by RT-PCR (data not shown). The use of GDNF and GFRα1 to trigger RET downstream pathways is in accordance with other previously published reports [36], [48] and also relies on the fact that GDNF is the strongest activator of RET signaling in enteric glial cells upon a number of physiological and pathological events[55]. Moreover, immune cells are expected to respond to RET ligands as they express the RET receptor, and GDNF and GFRα1 are known to be highly expressed in intestine during development[56]. These experimental evidence prompted us to hypothesize that during the course of intestinal inflammation in fetal or neonatal life, a clinical outcome often occurring in Hirschsprung patients and known as HAEC, RET^pos^ circulating immune cells from the periphery might interact with RET ligands present at the mucosal level. As a result of these possible interactions, immune cells could release pro-inflammatory mediators and actively contribute to intestinal inflammation.

The first group of genes clustered by our statistic analyses included molecules whose transcripts were modulated only following treatment of PBMCs with GDNF and GFRα1. We defined this cluster of genes as “RET-dependent”. Many of these genes encoded for chemokines or chemokine receptors, thus indicating that the engagement of RET might regulate the trafficking and the migration of immune cells upon inflammatory stimuli. Among these molecules, we found of particular interest the up-regulation of CCL20, an important mucosa-associated chemokine expressed in the intestinal epithelium and required for the recruitment of immune cells necessary for maintaining intestinal homeostasis[57], [58]. Indeed, CCL20, also known as or Macrophage Inflammatory Protein-3 (MIP3A) is a small cytokine strongly chemotactic for lymphocytes and dendritic cells in inflamed site upon binding on target cells the chemokine receptor 6 (CCR6)[59]. Therefore, it is likely that the engagement of RET at the mucosal sites (i.e. intestinal mucosa) is able to activate the CCL20-CCR6 pathway and, therefore, participate in modulating the homeostasis of the gut associated lymphoid tissue (GALT) in response to infections. Noteworthy, the expression of CCL20 has been found significantly up-regulated in PBMCs of patients with ulcerative colitis and Chron's Disease[60], [61], [62], being the role of CCL20 to link innate and adaptive immunity by attracting immature dendritic cells, effector memory T cells and B cells[59]. Moreover, CCL20 is also involved in activating the pro-inflammatory program in thyrocytes derived from the papillary thyroid carcinoma (PTC) where the fusion protein, termed the RET/PTC1 oncogene, displays ligand-independent autophosphorylation and has been shown to activate a set of genes *in vitro* and *in vivo* [37].

Within this first group of RET-dependent genes, we observed increased levels of CCL2 and IL-8 mRNA, along with the subsequent decreased amount of their receptor transcripts, CCR2 and IL-8Rα. Our data are in line with the experimental evidence showing that the binding of cytokines or chemokines to their putative membrane receptors trigger the internalization and degradation of the corresponding receptor, a phenomenon that has already been described for CCR2 and IL-8Rα [63], [64]. This could be a possible compensatory mechanism for regulating the RET-mediated lymphocyte trafficking to the inflammation site in GALT. Again, CCL2 and IL-8 are two important mediators on inflammation. Indeed, Chemokine (C–C motif) ligand 2 (CCL2), also known as monocyte chemotactic protein-1 (MCP-1), recruits monocytes, dendridic and T cells to sites of tissue injury[65], while IL-8 is a chemokine produced mainly by macrophages that mainly induces the chemotaxis of neutrophil granulocytes (IL-8 is also known as neutrophil chemotactic factor)[66].

Although not statistically significant, we observed that the degree of transcripts levels of the 13 RET dependent genes was generally higher in HSCR patients compared to healthy donors. Even the down-regulation of CCR2 and IL8-Rα was less pronounced in HSCR subjects compared to normal controls. This phenomenon suggests that the engagement of RET might induce a greater inflammatory responses in HSCR patients, in line with some clinical outcomes of the disease. The lack of statistic significance might be explained not only by the limited size of the sample, but also by the fact that none of the 6 HSCR patients analyzed was carrying a loss-of-function RET mutations (data not shown). We hypothesize that the stimulation of PBMCs from HSCR patients having pathogenic RET variants and whose lymphocytes express higher amounts of RET receptor might induce a more robust, and likely statistically significant, modulation of the 13 RET-dependent inflammatory genes compared to that observed in immune cells from healthy donors. We are currently enrolling through our clinical facility a larger numbers of patients affected by this rare diseases and carrying loss of functions RET mutations to validate out hypothesis. However, our data clearly show that the pattern of RET-dependent regulation of inflammatory genes is conserved in both healthy donors and HSCR patients. Therefore, it is conceivable that the stimulation of this tyrosine kinase in those≤20% of HSCR patients carrying a pathogenic RET mutation and an increased expression of RET receptor could lead to the onset of severe and aberrant inflammatory outcomes.

The second group of inflammatory genes (CSF-1R, IL1-R1, IL1-R2, TGF-β1, IL-18, IL-19, SPP1) was defined as “RET-independent”, because they were differentially expressed in PBMCs from HSCR patients and healthy donors regardless the stimulation of RET with its ligand and co-receptor. For this reason we also define them as “HSCR associated” genes. Among these genes, the ones that showed the most remarkable and statistically significant decreased levels of mRNA transcripts in HSCR patients compared to those of healthy donors were i) CSF-1R, also known as macrophage colony-stimulating factor receptor (M-CSFR or CD115) that controls the differentiation and function of macrophages[67], ii) IL1-R1 and IL1R2 that are involved in many cytokine-induced immune and inflammatory responses[68] and iii) TGF-β1 that belongs to TGF-β superfamily of cytokines and controls cell growth, proliferation and differentiation[69]. Since all these 4 genes are strongly involved also in regulating inflammatory processes also at mucosal levels, it is possible that the differences between HSCR patients and healthy donors in their transcript levels might represent the ground accounting for an immunological impairment predisposing to the onset of a life-threatening clinical outcomes occurring in HSCR patients. Indeed, HAEC, occurring in 5–30% of patients, is the most severe complication of HSCR and is characterized by explosive diarrhea, abdominal distension, fever and impending septic shock[70]. Although involvement of genetic and environmental factors, including local defense mechanisms and immune system dysfunction has been proposed, HAEC etiology remains mostly undisclosed[71], [72]. In this context, the down-regulation of pro-inflammatory genes, such as CSF-1R, IL1-R1, IL1-R2 and TGF-β1, in human primary immune cells could play an important role in the physiopathology of HAEC. As a matter of fact, studies performed in IL1-R1 knock-out mice provide evidence that the lack of IL1 receptor signaling pathway is associated with both an increased susceptibility to listeria monocytogenes and leishmania infections and with an attenuated delayed-type hypersensitivity responses[73]. Moreover, since macrophages are central effectors of innate immune responses for the recognition and clearance of pathogens[74], impairments in the CSF-1R transcription might be associated and/or contribute to the onset of HAEC. In addition, defects in TGF-β1, an important regulator of myeloid cell homeostasis[75], could potentially be linked to the onset of HAEC. Overall, a decreased expression of genes encoding for these important inflammatory mediators might be highly relevant to explain those clinical outcomes of HSCR diseases associated with dysfunctions of immune system.

Our experimental results also highlight that the vast majority of both RET-independent and RET-dependent genes are associated with the homeostasis and the functions of monocytes/macrophages (such as IL-1 associated genes, CSF-1R, CCL2, CCR2, CCL3, CCL4, CCL7, CCL20, CXCL1, CSF-1R, IL-6), neutrophils (such asIL-8, IL8-Rα, CXCL1), dendritic (such as CCL2, CCR2, CCL20) and NK (such as CCL4) cells. This suggests that the innate cellular arm of immune system play a major role in both the RET-mediated regulation of the immune system homeostasis and in the physiopathology of HSCR disease. Further studies are needed to specifically address the mechanisms and the effect of RET-induced activation of the several subsets of innate immune cells to possibly disclose their pathogenic role during the course of HSCR.

In summary, the present study links several genetic features of HSCR with its immunological counterparts by first showing that the increased expression of RET receptors on human PBMCs from HSCR patient correlates with the presence of loss of function RET mutations. Moreover, we demonstrate that the engagement of RET on primary immune cells induces the modulation of several genes involved in the inflammation processes. Remarkably, the regulation of these RET-dependent genes is present in both healthy donors and HSCR patients, although at different levels. Finally, our results identify another small set of RET-independent genes that, regardless of stimulation of RET with its ligand and co-receptor, are differently regulated in healthy donors versus HSCR patients. All together our experimental evidence show that the involvement of the immune system in the physiopathology of HSCR can be either RET dependent or independent.

## Materials and Methods

### Study Subjects

A cohort of 50 sporadic HSCR patients, 16 females and 34 males, recruited through regular hospital admission within the Department of Pediatric Surgery of Giannina Gaslini Institute (IGG), an Italian referential centre for HSCR disease, was studied. The diagnosis of HSCR was performed following all clinical and laboratory criteria, as recently revised by our group[76]. In particular, the examination of biological specimens of adequate rectal suction biopsies by the department of pathology included the Acetylcholinesterase (AChE), Lactate Dehidrogenase (LDH) and NADPH-diaphorase histochemical staining. In case of admitted patients that had already performed surgical procedures elsewhere, a comprehensive re-evaluation of gut specimen was repeated to avoid inclusion of non-HSCR patients. PBMCs were obtained from blood draws in accordance with the clinical protocol approved by the Institutional Review Board (IRB) of IGG. Parents of patients under 18 years old signed a consent form that was approved by the above mentioned IRBs in accordance with Italian laws and with Declaration of Helsinki. PBMCs from healthy volunteers were obtained from buffy coat of healthy donors that signed consent forms in accordance with clinical protocols approved by the IRB of Desio Hospital, Milan, Italy.

PBMCs were obtained by Ficoll-Hypaque (GE Healthcare) density gradient centrifugation from whole blood or buffy coats[77].

### Flow cytometry and confocal microscopy

For multicolour (up to 6 colours) (FACS Canto II, BD Pharmigen)[77], flow cytometric analyses PBMCs were stained with PerCP-Cy5-labeled CD14, pacific blue-labeled CD20 (Biolegend), allophycocyanin-labeled CD56 and fluorescein isothiocyanate-labeled CD3 (BD Pharmigen) monoclonal antibodies (mAbs). Viable cells were detected through the aqua live/dead staining (Invitrogen). For intracellular staining of RET, after an incubation of 30 minutes at 4°C with the above mentioned anti-CD56, -CD3, -CD20 and -CD14 mAbs, PBMCs were washed, fixed and permeabilized with cytofix/cytoperm following manufacturer's instructions (BD Pharmigen) and incubated separately for 30 minutes at 4°C with the phycoerythrin- labeled anti RET mAb (R&D System). To saturate and block FcγIII receptors on monocytes and avoid false positive, PBMCs were pre-incubated with human IgGs antibodies (Sigma-Aldrich) at 4°C for 20 minutes. Data were analyzed using FlowJo software (TreeStar) and the expression of RET receptor on immune cell subsets was measured by detecting the level of its mean fluorescence intensity (MFI).

For confocal microscopy, PBMCs were seeded on glass slides pre-treated with poly-L-lysine. After 30 minutes at 37°C, cells were washed in PBS added with 2% FBS (South American Origin; Lonza). Cells were fixed with paraformaldehyde (PFA) 4% in PBS for 15 minutes at room temperature (RT) on dark and washed with washing buffer [0,05% Tween 20 (Merckel) in PBS]. After blocking with 2% bovine serum albumin (BSA) for 30 minutes at RT, cells were stained with un-coniugated anti-RET pAb (Santa-Cruz) and anti-CD56 (MBL), or anti-CD19 (Dako) or anti-CD3 (Dako) Abs for one hour at RT. Before CD3 staining, cells were permeabilized with 0,3% Triton-X in PBS/2% FBS and incubated with blocking solution PBS/2%BSA in 5% donkey serum for one hour at RT. Cells were then washed and incubated with the secondary antibodies for 30 minutes at RT: Alexa Fluor® 488 Donkey anti-Rabbit, Alexa Fluor® 647 Donkey anti-Goat, Alexa Fluor® 488 Donkey anti-Goat and Alexa Fluor® 555 Donkey anti-Mouse (Invitrogen) were used. After washing, cells were stained with DAPI (Molecular Probes) for one minute on the dark to detect cell nucleus. Glasses were mounted with Fluor Preserve™ Reagent (Calbiochem) and cells were analyzed with a laser scanning confocal microscope (FluoView FV1000; Olympus). Images were acquired with an oil immersion objective (40×1.3 NA Plan-Apochromat; Olympus). For image analysis, Imaris X64 6.2.1 software (Bitplane, AG) was used.

### Culture of human cell lines

Neuroblastoma cell lines SK-N-MC and IMR-32 were cultured in Minimum Essential Medium Eagle MEM medium (SIGMA), monocytic leukemia cell line THP1 was grown in RPMI-1640 medium (Euroclone) and medullary thyroid carcinoma cell line MTC-TT was maintained in culture in F-12 Ham's medium (Euroclone). All medium were supplemented with 10% fetal bovine serum (FBS, New Zealand), 1% L-glutamine (100X), 100 U\ml penicillin and 100 mg\ml streptomycin and cell cultures were maintained at 37°C with 5% CO_2_ in a humidified incubator. Around 5X10E6 cells were collected from cell cultures: 3×10E6 cells were washed in DPBS and used for RNA extraction, 2×10E6 cells were used for cytofluorimetric experiments. All the above-mentioned cell lines have been purchased from ATCC, USA.

### RNA isolation and reverse transcription

Total RNA from cells was isolated by a commercial RNA purification kit (RNeasy Mini kit, Qiagen, GmbH, Germany) according to the manufacturer's protocol. 1 µg of total RNA was reverse transcribed by iScript cDNA synthesis kit (Bio-Rad Laboratories) according to the manufacturer's protocol. Real time quantitative PCR was performed using inventoried Assays-on-Demand™ provided by Applied Biosystems (Table 2). PCR reactions were performed using the iQ^TM^5 Real Time PCR (Bio-Rad Laboratories). The expression of mRNA in cell lines was evaluated using the comparative Ct method (ΔΔCt) and the normalization was achieved by the mean values of reference genes. Real Time PCR amplification was performed in triplicate and repeated twice.

**Table 2:** Assays for the real time PCR.

GENE NAME	ASSAY	GENE NAME	ASSAY
CCL2	Hs00234140_m1	IL8	Hs00174103_m1
CCL20	Hs00171125_m1	IL8-Rα	Hs00174304_m1
CCL3	Hs00234142_m1	PTGS2	Hs00153133_m1
CCL4	Hs00237011_m1	RET	Hs01120027_m1
CCL7	Hs00171147_m1	TNF	Hs00174128_m1
CCR2	Hs00356601_m1	POL2RF	Hs00222679_m1
CXCL1	Hs00236937_m1	GAPDH	Hs99999905_m1
IL1-β	Hs00174097_m1	RPLP0	Hs99999902_m1
IL6	Hs00985639_m1		

### Mutation Analysis

Genomic DNA was extracted from peripheral blood mononuclear cells (PBMCs) by a standard technique. DNA samples from HSCR patients were subjected to mutation screening of the twenty-one exons, and relative intron-exon boundaries of the *RET* gene by mean of direct sequencing of the corresponding amplification products. PCR products were purified by ExoSAP-IT (GE Healthcare) and directly sequenced using Big Dye v1.1 and a ABI3130 automated sequencer (Applied Biosystems, Foster City, CA, USA).

### Customized gene array analysis

10^7^ freshly purified PBMCs from three healthy donors and three HSCR patients without mutations of the RET coding sequence, were collected and resuspended in RPMI-1640. Samples were stimulated with human GDNF and human GFRα1 at the final concentration of 100ng/ml and 1 µg/ml respectively (R&D Systems) and incubated at 37°C in 5% CO_2_. After five hours incubation, cells were pelletted, RNA was extracted from cells and reverse transcribed as described above. For these samples, the detection of 96 genes was performed using custom Taqman Low Density Array (TLDA) cards (Applied Biosystem) on a 7900HT fast RT-PCR using SDS 2.2 software according to the manufacturer's protocols. Briefly 100ng of single-stranded cDNA was combined to TaqMan Universal PCR Master Mix 2X (Applied Biosystem) and water was added to a final volume of 100 µl per port. Thermal cycling conditions were the following: 50°C for 2 min, 95°C for 10 min, 40 cycles at 95°C for 15sec and 60°C for 1min. The expression level of each target gene was normalized on *GAPDH* and the analysis was carried out using the ΔCt method.

### Detection of soluble cytokines and chemokines in cell supernatants

After 5 hours of incubation with GDNF and GFRα1, cell supernatants were harvested, filtered using Costar Spin-X centrifuge tube 0.22 µm filters (Corning BV, Amsterdam, The Netherlands) and stocked at −80^0^C. All tested human proteins: CCL2, CCL3, CCL7, CCL20, CXCL1, IL-1β, CCL4, TNF, IL-6 and IL-8 were detected using commercially available R&D Duoset sandwich ELISA development systems according to the manufacture's instructions (R&D).

### Prediction analysis of the effects of RET mutations

The impact of amino acid changes detected in HSCR patients on the structure and function of the RET protein was predicted using the softwares like PolyPhen (Polymorphism Phenotyping; http://genetics.bwh.harvard.edu/pph/)[41], SIFT (Sorting Intolerant From Tolerant; http://sift.jcvi.org/www/SIFT_dbSNP.html)[42] and Panther (http://www.pantherdb.org)[43], [44]. ESEfinder v3.0 (http://rulai.cshl.edu/cgi-bin/tools/ESE3/esefinder.cgi?process=home) was used to investigate whether the nucleotide changes disrupted/created exonic splicing enhancers (ESEs) and/or branch or splice sites[45]. NNSPLICE V0.9 (http://www.fruitfly.org/seq_tools/splice.html) was also used to analyze intronic variants[46]. Default thresholds were adopted for all the softwares. In addition, literature data already available on functional assays performed on some of these mutations were also used to implement our prediction on *RET* mutations effects[8]. We then grouped all variants as pathogenic mutations or mild mutations.

### Statistical Analysis

RET MFIs values obtained by flow cytometry were analyzed by performing a non parametric Kruskal Wallis test to compare the levels of RET protein expressed on immune cell subsets.

Correlations between the amounts of the RET receptor and the levels of *RET* mRNA in specific cell lines (MTC-TT, SK-N-MC, THP1 and IMR32) were performed by using the Spearman rank test, while differences in the amounts of RET expression among the different genotypes were tested by the non parametric Kruskal Wallis test. Levels of RET protein have been compared between patients carrying pathogenic RET mutations and patients with a wild type RET genotype by performing the non parametric Mann-Whitney test.

Finally, differences in gene expression levels between HSCR patients and healthy donors, as assessed by the inflammatory CARD (TLDA), were analyzed using the ANOVA tests for repeated measures implemented in SPSS 17.1 (SPSS, Chicago, IL, USA). To further validate our analyses on the identified and candidate genes within the cohorts of 3 healthy donors and HSCR patients, non parametric tests were performed for those comparisons we found significant, namely 1) the Mann-Whitney test allowed to compare the six HSCR with the six controls for those genes found differently expressed in the two groups irrespectively of the treatment, 2) the Wilcoxon test was applied to the six samples treated toward the same sample at basal expression for those genes that were differently expressed following the treatment and not showing differences between cases and controls, and 3) the Mann-Whitney test allowed to assess differences of expression before and after GDNF treatment for those genes that were suspected to behave differently at the treatment in cases and in controls. We set the level of significance at p = 0.05.

Gene fold increases shown in Figure 5 are represented with 2-ddCt values, while raw data are included in Tables S3 and S4 are indicated as dCt values. This latter set of data have been then transformed in 2-ddCt (fold increase) by subtracting dCt of untreated PBMCs from dCt of treated PBMCs, thus obtaining the 2-ddCt values shown in Figure 5

## Supporting Information

Figure S1 **Confocal microscopy of RET receptor expressed on primary immune cells.** Representative examples from an healthy donor of fluorescent confocal microscopic images showing the expression of RET receptor on total PBMCs (panel A with RET labeled in green and cell nucleus in blue), NK cells (panel B with RET labeled in red, CD56 labeled in green and cell nucleus in blue), B cells (panel C with RET labeled in red, CD19 labeled in green and cell nucleus in blue), T cells (panel D with RET labeled in green, CD3 labeled in red and cell nucleus in blue). The co-localization of RET with the different markers of immune cells is labeled in yellow.
(TIF)

Figure S2 **Expression of RET on lymphocyte subsets associated with a single nucleotide polymorphism at exon 2.** Summary graph of statistical dot plots showing MFI values of RET receptor expressed on T (left), B (center) and NK (right) lymphocytes from 50 HSCR stratified on the basis of the genotype at the exon 2 of RET gene (SNP rs1800858). We did not detect any statistically significant association between the phenotypic distribution of RET receptor on immune cells with RET genotype at exon 2 SNP.
(TIF)

Figure S3 **Expression of RET on lymphocytes and monocytes associated with a single nucleotide polymorphism at exons 11,13 and 14.** Summary graph of statistical dot plots showing MFI values of RET receptor expressed on lymphocytes (left side of each panel) and monocytes (right side of each panel) from 50 HSCR stratified on the basis of the genotype at the exons 11 (left), 13 (center) and 14 (right) SNPs of RET gene. We did not detect any statistically significant association between the phenotypic distribution of RET receptor on immune cells with RET genotype at exons 11,13 and 14 SNPs.
(TIF)

Figure S4 **Expression of RET receptor on immune cells from HSCR patients associated with pathogenic and not pathogenic mutations of RET gene.** Summary graph of statistical histogram bars (upper panel) and dot plots (lower panel) showing MFI values of RET expressed on monocytes, T, B and NK lymphocytes from 50 HSCR patients stratified on the basis of individuals carrying pathogenic (gray histogram bars in upper panel and black symbols in the lower panel) or not pathogenic RET mutation (black histogram bars in upper panel and red symbols in the lower panel) or not carrying (empty histogram bars in upper panel and empty symbols in the lower panel) RET mutation at all.
(TIF)

Table S1 Prediction of RET mutation effects.
(DOCX)

Table S2 List of genes analyzed within the customized Taqman Low Density Array (TLDA) Card.
(DOCX)

Table S3 Analysis and statistical summary of all genes included in the customized Taqman Low Density Array Card.
(DOCX)

Table S4 Analysis and statistical summary of the 13 RET-dependent genes whose transcripts have been measured by real time qPCR to validate the results obtained by TLDA array.
(DOC)
