# Correction: Induction of RET Dependent and Independent Pro-Inflammatory Programs in Human Peripheral Blood Mononuclear Cells from Hirschsprung Patients

**DOI:** 10.1371/annotation/d3a96ff5-2a66-4454-8d8d-932ad4cfe906

**Published:** 2013-04-16

**Authors:** Marta Rusmini, Paola Griseri, Francesca Lantieri, Ivana Matera, Kelly L. Hudspeth, Alessandra Roberto, Joanna Mikulak, Stefano Avanzini, Valentina Rossi, Girolamo Mattioli, Vincenzo Jasonni, Roberto Ravazzolo, William J. Pavan, Alessio Pini-Prato, Isabella Ceccherini, Domenico Mavilio

Three authors were indicated as having the incorrect affiliations. These authors and their correct affiliations are as follows:

Marta Rusmini: Laboratory of Molecular Genetics, IRCCS, Giannina Gaslini Istitute, Genoa, Italy, Unit of Clinical and Experimental Immunology, Humanitas Clinical and Research Center, Rozzano, Milan, Italy

Ivana Matera: Laboratory of Molecular Genetics, IRCCS, Giannina Gaslini Istitute, Genoa, Italy, Unit of Clinical and Experimental Immunology, Humanitas Clinical and Research Center, Rozzano, Milan, Italy

Roberto Ravazzolo: Laboratory of Molecular Genetics, IRCCS, Giannina Gaslini Istitute, Genoa, Italy, DINOGMI Department, University of Genova, Genova, Italy 

