# Supplementary figures and images for: Induction of RET Dependent and Independent Pro-Inflammatory Programs in Human Peripheral Blood Mononuclear Cells from Hirschsprung Patients
Source: PLoS One. 2013 Mar 18;8(3):e59066. doi: 10.1371/journal.pone.0059066 (PMC3601093; doi:10.1371/journal.pone.0059066)

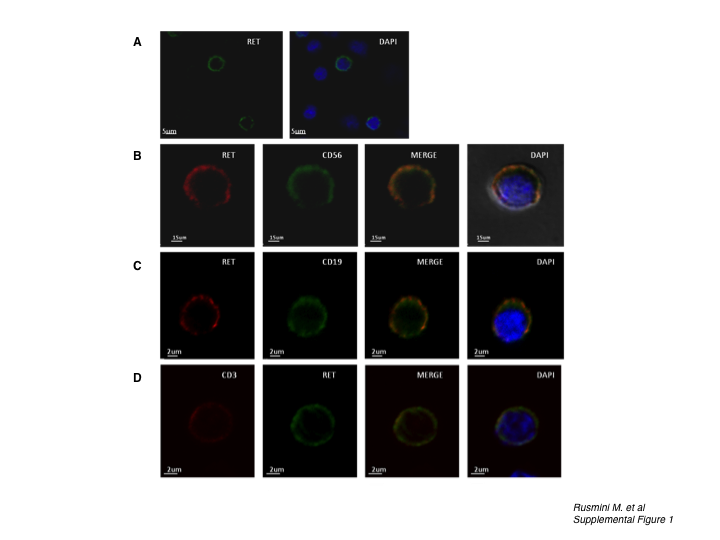

Supplement: Figure S1 — Confocal microscopy of RET receptor expressed on primary immune cells. Representative examples from an healthy donor of fluorescent confocal microscopic images showing the expression of RET receptor on total PBMCs (panel A with RET labeled in green and cell nucleus in blue), NK cells (panel B with RET labeled in red, CD56 labeled in green and cell nucleus in blue), B cells (panel C with RET labeled in red, CD19 labeled in green and cell nucleus in blue), T cells (panel D with RET labeled in green, CD3 labeled in red and cell nucleus in blue). The co-localization of RET with the different markers of immune cells is labeled in yellow. (TIF) [file pone.0059066.s001.tif]
