# Supplementary material for: Induction of RET Dependent and Independent Pro-Inflammatory Programs in Human Peripheral Blood Mononuclear Cells from Hirschsprung Patients
Source: PLoS One. 2013 Mar 18;8(3):e59066. doi: 10.1371/journal.pone.0059066 (PMC3601093; doi:10.1371/journal.pone.0059066)
Supplement: Table S1 — Prediction of RET mutation effects. (DOCX) [file pone.0059066.s005.docx]

**Supplemental Table 1**

**Prediction of RET mutation effects.**

| ***Nucleotide Change*** | ***Aminoacid***  ***Change*** | ***SNP database*** | ***Polyphen***  ***(PSIC)*** | ***SIFT*** | ***Panther*** | ***ESE***  ***Finder*** | ***SPL*** | ***Functional***  ***Test*** |
| --- | --- | --- | --- | --- | --- | --- | --- | --- |
| c.1394T>C | p.L465P | - | ++ (2.110) | not tolerated | n.a. |  |  |  |
| c.1759+1 G>A | - | - | - | - |  |  | no GT ss |  |
| c.1947G>A | p.S649S | - | - |  | silent mutation |  |  | Pathogenic (Gabriel 2002) |
| c.2314insGACC | Premature stop codon | - | - |  |  |  |  |  |
| c.3004A>G | p.S1002G | - | + (1.548) | tolerated | n.a |  |  |  |

**Legend**

The impact of amino acid changes on the structure and function of the RET protein was predicted using literature data and softwares like PolyPhen (Polymorphism Phenotyping; <http://genetics.bwh.harvard.edu/pph/>) (41), SIFT (Sorting Intolerant From Tolerant; <http://sift.jcvi.org/www/SIFT_dbSNP.html>) (42) and Panther (http://[www.pantherdb.org](http://www.pantherdb.org/)) (43, 44) . ESEfinder v3.0(<http://rulai.cshl.edu/cgi-bin/tools/ESE3/esefinder.cgi?process=home>) was used to investigate whether the nucleotide changes disrupted/created exonic splicing enhancers (ESEs) and/or branch or splice sites (45). NNSPLICE V0.9 (<http://www.fruitfly.org/seq_tools/splice.html>) was also used to analyze intronic variants (46). Default thresholds were used for all software.
