# Supplementary material for: Induction of RET Dependent and Independent Pro-Inflammatory Programs in Human Peripheral Blood Mononuclear Cells from Hirschsprung Patients
Source: PLoS One. 2013 Mar 18;8(3):e59066. doi: 10.1371/journal.pone.0059066 (PMC3601093; doi:10.1371/journal.pone.0059066)
Supplement: Table S2 — List of genes analyzed within the customized Taqman Low Density Array (TLDA) Card. (DOCX) [file pone.0059066.s006.docx]

**Supplemental Table 2**

**List of genes analyzed within the customized Taqman Low Density Array (TLDA) Card.**

| ***Gene Name*** | Assay |  | ***Gene Name*** | Assay |
| --- | --- | --- | --- | --- |
| ***ACTB*** | ACTB-Hs99999903_m1 |  | ***CXCL12*** | CXCL12-Hs00171022_m1 |
| ***B2M*** | B2M-Hs99999907_m1 |  | ***CXCL1*** | CXCL1-Hs00236937_m1 |
| ***18S*** | 18S-Hs99999901_s1 |  | ***CXCL9*** | CXCL9-Hs00171065_m1 |
| ***GAPDH*** | GAPDH-Hs00266705_g1 |  | ***CXCR3*** | CXCR3-Hs00171041_m1 |
| ***HPRT1*** | HPRT1-Hs99999909_m1 |  | ***CXCR4*** | CXCR4-Hs00237052_m1 |
| ***ALOX12*** | ALOX12-Hs00167524_m1 |  | ***CXCR6*** | CXCR6-Hs00174843_m1 |
| ***ALOX5*** | ALOX5-Hs00167536_m1 |  | ***CXCR7*** | CXCR7-Hs00604567_m1 |
| ***ANGPT1*** | ANGPT1-Hs00375822_m1 |  | ***FCGR3B-3A*** | FCGR3B;FCGR3A-Hs00275547_m1 |
| ***NOD2*** | NOD2-Hs00223394_m1 |  | ***GPR77*** | GPR77-Hs00218495_m1 |
| ***NOD1*** | NOD1-Hs00196075_m1 |  | ***IFNG*** | IFNG-Hs00174143_m1 |
| ***CCBP2*** | CCBP2-Hs00174299_m1 |  | ***IL10*** | IL10-Hs00174086_m1 |
| ***CCL11*** | CCL11-Hs00237013_m1 |  | ***IL12A*** | IL12A-Hs00168405_m1 |
| ***CCL13*** | CCL13-Hs00234646_m1 |  | ***IL12B*** | IL12B-Hs00233688_m1 |
| ***CCL15;***  ***CCL14*** | CCL15;CCL14-Hs00263142_m1 |  | ***IL13*** | IL13-Hs00174379_m1 |
| ***CCL16*** | CCL16-Hs00171123_m1 |  | ***IL15*** | IL15-Hs00542562_m1 |
| ***CCL17*** | CCL17-Hs00171074_m1 |  | ***IL17A*** | IL17A-Hs00174383_m1 |
| ***CCL18*** | CCL18-Hs00268113_m1 |  | ***IL18*** | IL18-Hs00155517_m1 |
| ***CCL19*** | CCL19-Hs00171149_m1 |  | ***IL19*** | IL19-Hs00604657_m1 |
| ***CCL1*** | CCL1-Hs00171072_m1 |  | ***IL1B*** | IL1B-Hs00174097_m1 |
| ***CCL20*** | CCL20-Hs00171125_m1 |  | ***IL1R1*** | IL1R1-Hs00991010_m1 |
| ***CCL21*** | CCL21-Hs00171076_m1 |  | ***IL1R2*** | IL1R2-Hs00174759_m1 |
| ***CCL22*** | CCL22-Hs00171080_m1 |  | ***IL1RN*** | IL1RN-Hs00174099_m1 |
| ***CCL25*** | CCL25-Hs00171144_m1 |  | ***IL23A*** | IL23A-Hs00372324_m1 |
| ***CCL26*** | CCL26-Hs00171146_m1 |  | ***IL24*** | IL24-Hs00169533_m1 |
| ***CCL2*** | CCL2-Hs00234140_m1 |  | ***IL2*** | IL2-Hs00174114_m1 |
| ***CCL3*** | CCL3-Hs00234142_m1 |  | ***IL4*** | IL4-Hs00174122_m1 |
| ***CCL4*** | CCL4-Hs99999148_m1 |  | ***IL4R*** | IL4R-Hs00166237_m1 |
| ***CCL5*** | CCL5-Hs00174575_m1 |  | ***IL6*** | IL6-Hs00174131_m1 |
| ***CCL7*** | CCL7-Hs00171147_m1 |  | ***IL8*** | IL8-Hs00174103_m1 |
| ***CCL8*** | CCL8-Hs00271615_m1 |  | ***IL8RA*** | IL8RA-Hs00174146_m1 |
| ***CCR1*** | CCR1-Hs00174298_m1 |  | ***IL8RB*** | IL8RB-Hs00174304_m1 |
| ***CCR2*** | CCR2;FLJ78302-Hs00356601_m1 |  | ***MIF*** | MIF-Hs00236988_g1 |
| ***CCR3*** | CCR3-Hs00266213_s1 |  | ***NOS2*** | NOS2-Hs00167257_m1 |
| ***CCR4*** | CCR4-Hs99999919_m1 |  | ***PTGS2*** | PTGS2-Hs00153133_m1 |
| ***CCR5*** | CCR5-Hs00152917_m1 |  | ***PTX3*** | PTX3-Hs00173615_m1 |
| ***CCR6*** | CCR6-Hs00171121_m1 |  | ***RARRES2*** | RARRES2-Hs00161209_g1 |
| ***CCR7*** | CCR7-Hs00171054_m1 |  | ***SIGIRR*** | SIGIRR-Hs00222347_m1 |
| ***CCR8*** | CCR8-Hs00174764_m1 |  | ***SPARC*** | SPARC-Hs00234160_m1 |
| ***CCRL1*** | CCRL1-Hs00356608_g1 |  | ***SPP1*** | SPP1-Hs00167093_m1 |
| ***CCRL2*** | CCRL2-Hs00243702_s1 |  | ***TGFB1*** | TGFB1-Hs00171257_m1 |
| ***CD14*** | CD14-Hs02621496_s1 |  | ***TLR2*** | TLR2-Hs00152932_m1 |
| ***CD68*** | CD68-Hs00154355_m1 |  | ***TLR3*** | TLR3-Hs00152933_m1 |
| ***CMKLR1*** | CMKLR1-Hs00356604_m1 |  | ***TLR4*** | TLR4-Hs00152939_m1 |
| ***CSF1*** | CSF1-Hs00174164_m1 |  | ***TLR9*** | TLR9-Hs00370913_s1 |
| ***CSF1R*** | CSF1R-Hs00234617_m1 |  | ***TNF*** | TNF-Hs00174128_m1 |
| ***CX3CL1*** | CX3CL1-Hs00171086_m1 |  | ***TNFRSF1A*** | TNFRSF1A-Hs00533568_g1 |
| ***CX3CR1*** | CX3CR1-Hs00365842_m1 |  | ***TNFRSF1B*** | TNFRSF1B-Hs00153550_m1 |
| ***CXCL10*** | CXCL10-Hs00171042_m1 |  | ***VEGFA*** | VEGFA-Hs00900054_m1 |
