# Supplementary material for: Induction of RET Dependent and Independent Pro-Inflammatory Programs in Human Peripheral Blood Mononuclear Cells from Hirschsprung Patients
Source: PLoS One. 2013 Mar 18;8(3):e59066. doi: 10.1371/journal.pone.0059066 (PMC3601093; doi:10.1371/journal.pone.0059066)
Supplement: Table S3 — Analysis and statistical summary of all genes included in the customized Taqman Low Density Array Card. (DOCX) [file pone.0059066.s007.docx]

**Supplemental Table 3**

**Analysis and statistical summary of all genes included in the customized Taqman Low Density**

**Array Card**

|  | ***Healthy Donor PBMCs***  ***Treated - Mean (95%CI)*** | ***Healthy Donor PBMCs***  ***Untreated - Mean (95%CI)*** | ***HSCR PBMCs***  ***Treated - Mean (95%CI)*** | ***HSCR PBMCs***  ***Untreated - Mean (95%CI)*** | ***Group*** |
| --- | --- | --- | --- | --- | --- |
| ***CCL2*** | 6.040 (3.531; 8.549) | 8.740 (5.420; 12.060) | 4.961 (3.952; 5.971) | 7.205 (3.961; 10.448) | Group 1 |
| ***CCL20*** | 6.641 (2.914; 10.367) | 7.619 (3.643; 11.595) | 6.268 (3.316; 9.220) | 8.768 (2.658; 14.877) | Group 1 |
| ***CCL3*** | 2.953 (0.731; 5.174) | 4.331 (1.090; 7.573) | 1.323 (-2.222; 4.868) | 3.465 (-3.435; 10.365) | Group 1 |
| ***CCL4*** | 3.617 (2.636; 4.599) | 4.343 (2.972; 5.714) | 2.135 (-0.009; 4.279) | 3.309 (-0.338; 6.956) | Group 1 |
| ***CCL7*** | 7.936 (3.035; 12.837) | 9.606 (7.688; 11.524) | 7.214 (6.443; 7.985) | 9.106 (7.856; 10.356) | Group 1 |
| ***CCR2*** | 5.007 (3.922; 6.091) | 3.846 (1.505; 6.187) | 6.217 (2.959; 9.475) | 5.537 (3.060; 8.014) | Group 1 |
| ***CXCL1*** | 4.828 (1.831; 7.826) | 6.236 (3.620; 8.853) | 4.418 (2.379; 6.457) | 7.399 (1.737; 13.061) | Group 1 |
| ***IL1B*** | 0.649 (-2.669; 3.967) | 1.876 (-1.843; 5.596) | -0.202 (-1.762; 1.359) | 2.672 (-2.377; 7.722) | Group 1 |
| ***IL6*** | 11.161 (8.237; 14.086) | 11.805 (11.059; 12.552) | 9.043 (6.681; 11.406) | 11.538 (7.700; 15.376) | Group 1 |
| ***IL8*** | -0.478 (-1.861; 0.905) | 0.442 (-0.904; 1.788) | -1.577 (-1.929; -1.225) | -0.317 (-1.424; 0.791) | Group 1 |
| ***IL8RA*** | 11.174 (7.942; 14.406) | 9.564 (8.149; 10.980) | 9.417 (4.847; 13.988) | 8.768 (3.982; 13.553) | Group 1 |
| ***PTGS2*** | 6.439 (4.139; 8.740) | 7.016 (4.091; 9.941) | 5.446 (3.767; 7.125) | 7.252 (3.390; 11.114) | Group 1 |
| ***TNF*** | 5.844 (5.378; 6.311) | 6.190 (5.761; 6.619) | 5.151 (1.351; 8.952) | 6.346 (2.949; 9.743) | Group 1 |
| ***CSF1R*** | 3.480 (3.017; 3.943) | 3.169 (2.776; 3.563) | 4.866 (2.790; 6.942) | 4.409 (3.585; 5.232) | Group 2 |
| ***IL18*** | 8.678 (7.188; 10.168) | 8.437 (6.562; 10.313) | 9.249 (8.041; 10.458) | 10.411 (8.596; 12.226) | Group 2 |
| ***IL19*** | 10.373 (8.976; 11.771) | 9.715 (8.055; 11.375) | 8.558 (7.074; 10.041) | 8.809* | Group 2 |
| ***IL1R1*** | 3.531 (1.945; 5.117) | 3.005 (2.498; 3.511) | 6.046 (4.680; 7.411) | 5.857 (4.934; 6.780) | Group 2 |
| ***IL1R2*** | 4.536 (3.473; 5.599) | 4.125 (3.405; 4.845) | 7.739 (3.763; 11.714) | 7.186 (5.157; 9.216) | Group 2 |
| ***SPP1*** | 9.326 (6.216; 12.435) | 9.969 (8.661; 11.277) | 6.516 (3.497; 9.534) | 7.130 (4.514; 9.746) | Group 2 |
| ***TGFB1*** | -1.574 (-2.173; -0.976) | -1.848 (-2.060; -1.636) | -0.494 (-1.244; 0.256) | -0.337 (-1.003; 0.329) | Group 2 |
| ***ANGPT1*** | 10.868 (8.907; 12.828) | 9.015 (8.260; 9.769) | 10.987 (8.899; 13.074) | 11.043 (7.770; 14.316) | Group 3 |
| ***CCL22*** | 3.762 (0.920; 6.604) | 3.610 (0.706; 6.514) | 5.218 (1.300; 9.135) | 6.057 (2.444; 9.671) | Group 3 |
| ***CCR4*** | 3.389 (2.751; 4.027) | 3.094 (2.454; 3.735) | 4.025 (2.693; 5.358) | 4.160 (2.826; 5.494) | Group 3 |
| ***CCR6*** | 8.064 (7.373; 8.755) | 7.524 (6.999; 8.049) | 7.014 (6.248; 7.780) | 7.087 (6.575; 7.599) | Group 3 |
| ***CXCR3*** | 7.009 (4.748; 9.269) | 6.551 (4.023; 9.078) | 7.211 (4.824; 9.599) | 7.175 (4.704; 9.646) | Group 3 |
| ***IL23A*** | 7.257 (5.939; 8.575) | 6.844 (5.135; 8.554) | 6.654 (5.859; 7.449) | 6.951 (6.282; 7.620) | Group 3 |
| ***IL8RB*** | 9.336 (8.388; 10.284) | 7.947 (6.432; 9.462) | 8.006 (4.747; 11.264) | 8.043 (5.207; 10.879) | Group 3 |
| ***NOD1*** | 6.858 (5.494; 8.221) | 6.722 (5.660; 7.784) | 6.569 (4.643; 8.496) | 7.034 (4.689; 9.378) | Group 3 |
| ***TLR2*** | 4.410 (3.184; 5.637) | 4.610 (4.264; 4.956) | 3.052 (1.873; 4.230) | 3.774 (2.234; 5.314) | Group 3 |
| ***TNFRSF1A*** | 2.076 (0.488; 3.665) | 1.719 (-0.011; 3.449) | 2.990 (0.323; 5.657) | 2.995 (0.634; 5.356) | Group 3 |
| ***CCL1*** |  | 12.865* | 14.435* |  | n.d. |
| ***CCL19*** | 15.401* | 11.966° (-0.180; 24.113) | 11.218* | 11.378* | n.d. |
| ***CCL25*** | 15.561* |  | 12.298 (-10.690; 35.286)° |  | n.d. |
| ***CCL8*** | 12.383 (9.718; 15.047) ° | 14.853* | 12.098 (3.355; 20.841)° | 14.168* | n.d. |
| ***CX3CL1*** |  |  |  |  | n.d. |
| ***CXCL12*** |  |  |  | 13.409* | n.d. |
| ***IL1RN*** | 11.878 (6.892; 16.865)° | 14.246* | 12.732 (10.033; 15.431) | 9.696* | n.d. |
| ***NOS2*** | 12.504* | 12.589 (0.396; 24.783)° | 12.577* | 13.877* | n.d. |
| ***RARRES2*** |  | 12.029 (2.292; 21.766)° | 11.922 (5.201; 18.642)° | 12.116 (10.673; 13.560) | n.d. |
| ***ALOX12*** | 8.963 (7.425; 10.501) | 8.241 (5.201; 11.281) | 9.183 (5.337; 13.030) | 8.826 (6.819; 10.833) | n.s. |
| ***ALOX5*** | 1.309 (-1.417; 4.035) | 0.732 (-1.061; 2.526) | 2.772 (0.862; 4.683) | 2.659 (1.207; 4.111) | n.s. |
| ***CCL5*** | 1.748 (0.078; 3.417) | 1.629 (0.262; 2.996) | 1.702 (0.023; 3.381) | 1.796 (0.680; 2.911) | n.s. |
| ***CCR1*** | 4.174 (3.594; 4.754) | 4.293 (2.521; 6.064) | 4.353 (1.073; 7.634) | 4.400 (1.757; 7.043) | n.s. |
| ***CCR3*** | 6.957 (5.020; 8.894) | 6.530 (4.110; 8.951) | 7.861 (4.640; 11.083) | 7.638 (4.999; 10.278) | n.s. |
| ***CCR5*** | 6.099 (4.502; 7.697) | 5.594 (4.127; 7.062) | 6.404 (5.041; 7.766) | 6.475 (5.231; 7.718) | n.s. |
| ***CCR7*** | 3.335 (0.983; 5.688) | 2.967 (0.644; 5.289) | 2.880 (0.997; 4.764) | 2.782 (1.554; 4.010) | n.s. |
| ***CCR8*** | 9.816 (5.907; 13.726) | 10.319 (5.690; 14.948) | 11.569 (7.349; 15.789) | 11.853 (-4.949; 28.656)° | n.s. |
| ***CCRL1*** | 10.153 (5.806; 14.500) | 9.305 (7.628; 10.982) | 12.013 (9.634; 14.392) | 11.615 (5.159; 18.070) | n.s. |
| ***CCRL2*** | 5.806 (3.406; 8.205) | 6.082 (2.872; 9.292) | 5.361 (4.138; 6.584) | 5.220 (4.200; 6.240) | n.s. |
| ***CD14*** | 1.482 (0.561; 2.403) | 1.380 (0.028; 2.733) | 2.148 (-0.093; 4.389) | 2.388 (1.324; 3.453) | n.s. |
| ***CD68*** | 1.367 (0.278; 2.457) | 1.288 (0.992; 1.583) | 1.946 (-0.821; 4.714) | 1.706 (-0.217; 3.629) | n.s. |
| ***CSF1*** | 6.935 (4.232; 9.639) | 6.886 (5.753; 8.019) | 6.962 (2.824; 11.100) | 7.400 (5.169; 9.632) | n.s. |
| ***CX3CR1*** | 6.878 (4.933; 8.823) | 6.896 (4.429; 9.363) | 5.985 (3.532; 8.438) | 6.325 (3.916; 8.733) | n.s. |
| ***CXCL10*** | 9.850 (9.532; 10.168) | 9.481 (8.540; 10.422) | 8.011 (-1.366; 17.388) | 7.654 (-0.398; 15.707) | n.s. |
| ***CXCL9*** | 11.578 (-2.164; 25.320)° | 10.915 (9.438; 12.391) | 8.480 (-1.615; 18.574)° | 8.326° (1.268; 15.384) | n.s. |
| ***CXCR4*** | 0.976 (-0.512; 2.464) | 0.470 (-1.026; 1.966) | 0.854 (-0.536; 2.245) | 0.511 (-0.638; 1.659) | n.s. |
| ***CXCR6*** | 8.601 (3.812; 13.390) | 7.873 (3.899; 11.847) | 7.849 (5.492; 10.207) | 7.743 (5.500; 9.986) | n.s. |
| ***CXCR7*** | 7.659 (2.073; 13.245) | 6.819 (3.304; 10.334) | 9.432 (7.522; 11.342) | 9.947 (6.646; 13.249) | n.s. |
| ***FCGR3B-3A*** | 2.298 (0.046; 4.549) | 1.764 (1.172; 2.355) | 1.929 (-0.990; 4.848) | 1.642 (-0.266; 3.551) | n.s. |
| ***GPR77*** | 5.477 (3.187; 7.767) | 4.812 (2.716; 6.907) | 6.989 (0.417; 13.562) | 6.438 (1.646; 11.230) | n.s. |
| ***IFNG*** | 10.410 (7.333; 13.487) | 9.489 (9.322; 9.656) | 8.940 (2.621; 15.258) | 9.177 (2.696; 15.657) | n.s. |
| ***IL10*** | 7.604 (6.327; 8.881) | 7.759 (6.568; 8.950) | 6.572 (4.413; 8.731) | 7.928 (5.306; 10.550) | n.s. |
| ***IL12A*** | 10.814 (7.388; 14.241) | 10.879 (10.102; 11.656) | 11.794 (8.170; 15.417) | 11.511 (5.998; 17.024)° | n.s. |
| ***IL15*** | 7.790 (5.772; 9.808) | 7.155 (4.607; 9.702) | 7.063 (1.150; 12.975) | 7.511 (3.347; 11.675) | n.s. |
| ***IL2*** | 11.400 (7.017; 15.784) | 11.205 (10.187; 12.223) | 13.147 (-10.368; 36.662)° | 13.354* | n.s. |
| ***IL24*** | 9.208 (7.361; 11.054) | 7.954 (7.205; 8.704) | 9.429 (4.147; 14.712) | 8.940 (6.165; 11.715) | n.s. |
| ***IL4*** | 13.237 (9.768; 16.707) | 13.248 (11.300; 15.196) | 14.083 (2.887; 25.279)° | 13.850 (12.450; 15.249)° | n.s. |
| ***IL4R*** | 1.878 (1.327; 2.430) | 1.521 (1.295; 1.748) | 2.440 (1.679; 3.200) | 2.376 (0.588; 4.165) | n.s. |
| ***MIF*** | 2.506 (1.125; 3.887) | 2.379 (0.696; 4.063) | 1.491 (0.730; 2.253) | 1.455 (0.754; 2.156) | n.s. |
| ***NOD2*** | 4.760 (2.686; 6.833) | 4.406 (2.092; 6.719) | 5.942 (2.875; 9.010) | 6.034 (3.032; 9.036) | n.s. |
| ***PTX3*** | 7.797 (5.792; 9.801) | 8.036 (6.301; 9.771) | 7.476 (3.059; 11.894) | 8.635 (4.960; 12.311) | n.s. |
| ***SIGIRR*** | 3.921 (2.268; 5.573) | 3.760 (2.996; 4.524) | 4.034 (1.322; 6.747) | 3.961 (0.175; 7.747) | n.s. |
| ***SPARC*** | 5.730 (4.115; 7.345) | 5.364 (2.535; 8.193) | 6.460 (2.742; 10.178) | 6.018 (2.293; 9.743) | n.s. |
| ***TLR3*** | 9.816 (1.890; 17.743)° | 8.565 (-2.168; 19.298)° | 12.072 (7.446; 16.698)° | 13.090 (-3.894; 30.074)° | n.s. |
| ***TLR4*** | 4.826 (3.195; 6.457) | 4.310 (3.769; 4.851) | 5.644 (2.014; 9.274) | 5.695 (2.970; 8.419) | n.s. |
| ***TLR9*** | 5.361 (2.388; 8.334) | 4.972 (3.207; 6.737) | 5.464 (4.569; 6.360) | 5.771 (5.014; 6.527) | n.s. |
| ***TNFRSF1B*** | 2.070 (0.781; 3.358) | 1.561 (0.985; 2.138) | 1.967 (-0.828; 4.762) | 1.883 (-0.571; 4.336) | n.s. |
| ***VEGFA*** | 1.015 (-3.307; 5.337) | 1.189 (-3.570; 5.948) | 2.615 (-0.454; 5.684) | 2.389 (-1.033; 5.812) | n.s. |

**Legend**

The table shows the modulation of all PBMC genes (mean ΔCt values) after treatment with GDNF and GFRα1 with mean values and the relative confidence interval (95%). The statistical analyses divided all genes in many subgroups: *a)* Group 1 includes the RET-dependent genes; *b)* Group 2 includes the RET-independent genes; *c)* Group 3 includes genes differently modulated upon treatment with GDNF and GFRα1 within each individual of both cohorts of healthy donors and HSCR patients; *d)* The statement n.d. (not determined) is referred to those genes for which statistical analyses was not possible (missing data); *e)* The statement n.s. is referred to those gene for which we did not detect any statistically significant difference in their mRNA transcript levels (* = data for one sample only) (Refer to the manuscript text for details).
