# Supplementary material for: Induction of RET Dependent and Independent Pro-Inflammatory Programs in Human Peripheral Blood Mononuclear Cells from Hirschsprung Patients
Source: PLoS One. 2013 Mar 18;8(3):e59066. doi: 10.1371/journal.pone.0059066 (PMC3601093; doi:10.1371/journal.pone.0059066)
Supplement: Table S4 — Analysis and statistical summary of the 13 RET-dependent genes whose transcripts have been measured by real time qPCR to validate the results obtained by TLDA array. (DOC) [file pone.0059066.s008.doc]

**Supplemental Table 4**

**Analysis and statistical summary of the 13 RET-dependent genes whose transcripts have been measured by real time qPCR to validate the results obtained by TLDA array.**

|  | ***Healthy Donor PBMCs***  ***Treated – Mean (95%CI )*** | ***Healthy Donor PBMCs***  ***Untreated - Mean (95%CI)*** | ***HSCR PBMCs***  ***Treated - Mean (95%CI)*** | ***HSCR PBMCs***  ***Untreated - Mean (95%CI)*** | ***Group*** |
| --- | --- | --- | --- | --- | --- |
| ***CCL2*** | 7.535 (6.06; 9.01) | 7.557 (5.71; 9.41) | 5.353 (-0.99; 11.70)) | 6.613 (0.88; 12.35) | Group 1 |
| ***CCL20*** | 8.722 (7.95; 9.49) | 9.366 (7.69; 11.04) | 8.802 (3.28; 14.32) | 9.503 (5.06; 13.95) | Group 1 |
| ***CCL3*** | 7.862 (4.47; 11.25) | 8.031 (4.91; 11.15) | 5.283 (1.92; 8.65) | 5.965 (3.49; 8.44) | Group 1 |
| ***CCL4*** | 4.956 (3.14; 6.77) | 4.954 (2.82; 7.09) | 2.762 (-0.74; 6.26) | 3.492 (0.34; 6.65) | Group 1 |
| ***CCL7*** | 9.137 (8.68; 9.60) | 9.097 (6.91; 11.28) | 7.022 (-1.00; 15.05) | 8.303 (2.53; 14.07) | Group 1 |
| ***CCR2*** | 6.354 (5.94; 6.77) | 6.283 (4.51; 8.06) | 7.773 (7.09; 8.46) | 7.081 (6.48; 7.68) | Group 1 |
| ***CXCL1*** | 6.789 (5.15; 8.43) | 7.304 (4.63; 9.98) | 6.187 (-0.59; 12.96) | 6.458 (1.63; 11.28) | Group 1 |
| ***IL1B*** | 4.454 (2.46; 6.45) | 4.468 (2.25; 6.69) | 2.769 (-2.82; 8.36) | 3.150 (-1.76; 8.06) | Group 1 |
| ***IL6*** | 11.010 (7.82; 14.20) | 10.845 (7.67; 14.02) | 8.262 (4.47; 12.05) | 9.174 (4.33; 14.02) | Group 1 |
| ***IL8*** | 1.320 (0.36; 2.28) | 1.663 (-2.16; 5.49) | 0.075 (-2.88; 3.03) | 0.094 (-1.92; 2.11) | Group 1 |
| ***IL8RA*** | 6.050 (1.62; 10.48 ) | 6.180 (2.44; 9.92) | 7.456 (3.22; 11.69) | 7.056 (3.16; 10.95) | Group 1 |
| ***PTGS2*** | 1.586 (-24.19; 27.37) | 9.353 (4.34; 14.37) | -0.137 (-23.82; 23.54) | 5.851 (2.37; 9.33) | Group 1 |
| ***TNF*** | 7.569 (5.55; 9.59) | 7.177 (4.15; 10.20) | 6.689 (2.38; 11.00) | 6.804 (2.77; 10.84) | Group 1 |

**Legend**

The table shows the modulation of all 13 RET-dependent genes (mean ΔCt values) after treatment of PBMCs with GDNF and GFRα1 with mean values obtained by qPCR and the relative confidence interval (95%). Data from each gene is reported as mean of the three samples. All values are normalized on the GAPDH gene expression.
